# Phase contrast-derived cerebral blood flow is associated with neurodegeneration and cerebrovascular injury in older adults

**DOI:** 10.3389/fnins.2025.1538956

**Published:** 2025-07-04

**Authors:** Jeffrey D. Pyne, Clarissa D. Morales, A. Zarina Kraal, Mohamad J. Alshikho, Patrick J. Lao, Indira C. Turney, Erica Amarante, Rafael V. Lippert, Julia F. Chang, Jose Gutierrez, Jennifer J. Manly, Richard Mayeux, Adam M. Brickman

**Affiliations:** ^1^Taub Institute for Research on Alzheimer's Disease and the Aging Brain, Vagelos College of Physicians and Surgeons, Columbia University, New York, NY, United States; ^2^Gertrude H. Sergievsky Center, Vagelos College of Physicians and Surgeons, Columbia University, New York, NY, United States; ^3^Department of Neurology, Vagelos College of Physicians and Surgeons, Columbia University, New York, NY, United States; ^4^Department of Epidemiology, Mailman School of Public Health, Columbia University, New York, NY, United States

**Keywords:** cerebral blood flow, aging, cerebrovascular, phase contrast MRI, hypoperfusion, white matter

## Abstract

Global cerebral blood flow and the local delivery of blood through the vascular network are essential to maintain brain and cognitive health throughout the lifespan. In this cross-sectional study, we examined the association of extracranial blood flow into the brain, measured with phase contrast magnetic resonance imaging, with regional brain volumes, cortical thickness, white matter tract integrity, white matter hyperintensity volume, and cerebral microbleeds. Our study included 311 older adults (mean age: 77 years, standard deviation: 5.6) from the Washington Heights Inwood Columbia Aging Project (WHICAP), a community-based study in northern Manhattan. We found that lower extracranial cerebral blood flow is associated with lower cortical regional volumes, lower white matter tract integrity, and higher white matter hyperintensity volume. We observed that lower extracranial cerebral blood flow, quantified by total, anterior, and posterior circulations, is associated with lower white matter tract integrity in the forceps minor, cingulum cingulate gyrus, and inferior fronto-occipital fasciculus. Additionally, lower total extracranial cerebral blood flow is associated with higher white matter hyperintensity volume, a marker of small vessel cerebrovascular disease. These findings support our hypothesis that lower extracranial cerebral blood flow is associated with a greater degree of vascular brain injury and indicators of neurodegeneration and are consistent with the guiding conceptual framework that diminished extracranial blood flow could be a factor that promotes or exacerbates neurodegeneration and cerebrovascular injury in older adults. Future longitudinal studies are needed to establish causality and temporality.

## 1 Introduction

Cerebral blood flow supply to the perfusion-sensitive brain is an essential factor in maintaining overall brain tissue structure, health, and function throughout the lifespan (Ginsberg, 2016). Measures of regional cerebral volumes, cortical thickness, white matter tract integrity, white matter hyperintensity volume, and cerebral microbleeds reflect aspects of brain health that are implicated in cognitive aging and dementia (MacDonald and Pike, 2021; Caunca et al., 2019). The brain is a hemodynamically complex organ with both extracranial and intracranial vascular factors that influence how blood is circulated throughout its tissue (Meng et al., 2015; Fisher et al., 2022; Thore et al., 2007; Stefanidis et al., 2019; Shabir et al., 2018; Bia et al., 2011). Extracranial vascular mechanisms pertain to systemic factors that influence blood flow of the major arteries, at the level of the neck and Circle of Willis, that supply the brain, while intracranial vascular factors pertain to regional blood flow distribution at the level of pial arteries down to the arteriole-capillary vascular bed. Total cerebral blood flow could represent or be affected by the combined influence of all extracranial and intracranial vascular factors. The relationship between total cerebral blood flow and other markers of brain health could be bidirectional; for example, lower total blood flow supply could have downstream impact on brain regions and atrophy of brain regions could decrease the overall supply needs of the brain. It is essential to examine total cerebral blood flow, as it may independently fail to meet the metabolic demands of the brain, influencing both brain structure and function, as well as regional blood flow patterns. While numerous studies examined cross-sectional and longitudinal associations between intracranially-derived total cerebral blood flow with markers of brain health (Bahrani et al., 2017; Bastos-Leite et al., 2008; Chen et al., 2013; Staffaroni et al., 2019), the relationship between extracranially-derived cerebral blood flow and markers of neurodegeneration and cerebrovascular injury remains unclear. In theory, measurement of total cerebral blood flow via extracranial or intracranial approaches should be equivalent, but practical methodological limitations with current techniques could lead to bias measurements of flow and inaccurate conclusions. In the current study, we used phase contrast magnetic resonance imaging (MRI) to quantify extracranial cerebral blood flow and investigate its association with markers of small vessel cerebrovascular disease and neurodegeneration among racially and ethnically diverse older adults.

Phase contrast MRI provides a measure of absolute cerebral blood flow with minimal methodological assumptions (Zananiri et al., 1991; Koerte et al., 2013) and is thus practical for cross-sectional and longitudinal studies in older adults with varying degrees of cerebrovascular disease. Other MRI-based blood flow measures, such as those commonly derived with pseudo-continuous or pulsed arterial-spin labeling (ASL), are often used to measure total cerebral blood flow in aging or disease states, but these methods alone cannot measure total blood flow without bias (Wu et al., 2010; Aslan et al., 2010; Jung et al., 2010). Phase contrast imaging is more accurate for absolute flow quantification than most routine velocity- or flow-based measurement techniques, such as transcranial doppler (TCD) or pseudo-continuous ASL, especially in conditions where blood flow can deviate from the idealized behavior assumptions. For example, TCD measures blood vessel flow velocity, which relies upon an axisymmetric flow-profile assumption to estimate flow rate (Mynard and Steinman, 2013), an assumption that may not be valid in the context of cerebrovascular disease and aging (Gelfand et al., 2006). Similarly, pseudo-continuous ASL-based measurements are dependent on the velocity of incoming arterial flow, both in terms of MR tagging efficiency (Wu et al., 2010; Aslan et al., 2010) and in cases of arterial transit artifacts (Ozpar et al., 2023), which can affect the accuracy of total blood flow quantification. Overall, intracranial measurements of total cerebral blood flow commonly derived from pseudo-continuous ASL are susceptible to bias, including variability in velocity tagging efficiency and partial volume effects. However, advanced ASL techniques, such as multi-phase or velocity-selective ASL, can overcome these limitations (Jung et al., 2010; Wong et al., 2006). Measurements of total cerebral blood flow obtained through extracranially-based phase contrast imaging offers reduced susceptibility to bias, making it particularly adventitious for longitudinal studies or clinical trials designed to measure within-participant changes in flow, but is also useful across cross-sectional studies where high variance of cerebral flow rates are expected amongst the cohort. Therefore, for older adults with varying degrees of cerebrovascular injury, phase contrast is one of the ideal tools to quantify absolute flow measurements and to investigate the influence of extracranial cerebral blood flow on brain health.

In the current cross-sectional study, we examined the association of extracranial cerebral blood flow, sectioned into total, anterior, and posterior circulation, with measures of brain health, including regional cerebral volumes, cortical thickness, white matter tract integrity, white matter hyperintensity volume, and cerebral microbleeds in older adults from a racially and ethnically diverse community-based cohort in New York City. We hypothesized that lower extracranial cerebral blood flow is associated with a greater degree of vascular injury and indicators of neurodegeneration. We formed our hypothesis on the guiding conceptual framework that diminished extracranial blood flow could be a factor that promotes or exacerbates neurodegeneration and cerebrovascular injury in older adults. We expected to observe more pronounced associations in brain regions with particularly vulnerable vascular supply, and thus greater susceptibility to chronic hypoperfusion, such as in deep white matter.

## 2 Materials and methods

### 2.1 Participants

Participants were selected from the Washington Heights-Inwood Columbia Aging Project (WHICAP), an ongoing community study of cognitive aging and dementia that enrolls older adults (65+) residing in northern Manhattan, New York (Turney et al., 2022; Avila et al., 2021; Brickman et al., 2021). In 2019, phase contrast imaging was added to the ongoing WHICAP brain MRI study, and participants scanned with this technique between 2019 and 2024 were included in the study subset. Participants undergo clinical assessments at longitudinal visits approximately every 2 years (Turney et al., 2022; Avila et al., 2021; Brickman et al., 2021). The WHICAP cohort comprises the three major race and ethnicity groups that characterize the community surrounding Columbia University Irving Medical Center, including non-Hispanic White (White), non-Hispanic Black (Black), and Hispanic (Latinx) participants. Participants self-report race and ethnicity. Common vascular risk factors, selected based on their risk for influencing the vascular health and blood flow of the brain, were ascertained by self-reported diagnosis of hypertension, diabetes, and heart disease (arrhythmias, coronary artery disease, and/or congestive heart failure) (Luchsinger et al., 2005). Each vascular risk factor was coded as 0 for absent or 1 for present. These dichotomous variables were summed for a vascular risk factor score that ranged from 0 to 3. This study was approved by the institutional review board at Columbia University Irving Medical Center; all participants gave written informed consent.

### 2.2 MRI acquisition

Participants were scanned on a General Electric Medical Systems SIGNA Premier 3 T MRI scanner with a 64-channel head coil. MRI scanning included phase contrast, vascular angiography, T1-weighted, T2-weighted, diffusion tensor, arterial spin labeling, and susceptibility weighted imaging sequences. The phase contrast sequence was used for extracranial flow-rate measurements of the blood vessels supplying the brain, including the internal carotid and vertebral arteries (Zarrinkoob et al., 2015; Wu et al., 2016). The vascular angiography non-contrast time of flight (TOF) sequence measured vascular anatomy leading to the brain and helped with positioning of the phase contrast sequence (Zarrinkoob et al., 2015). The T1-weighted magnetization-prepared rapid acquisition gradient echo (MPRAGE) sequence was used to measure regional volumes and cortical thickness. The T2-weighted fluid-attenuated inversion recovery (FLAIR) sequence was used to measure white matter hyperintensity (WMH) volume, a marker of small vessel cerebrovascular disease (Wardlaw et al., 2019). Diffusion tensor imaging (DTI) was used to measure white matter microstructure integrity (Soares et al., 2013). The susceptibility weighted imaging (SWI) sequence was used to detect and count cerebral microbleeds (Greenberg et al., 2009). Arterial spin labeling (ASL) was used to measure intracranially-derived total cerebral blood flow for comparison against phase contrast extracranially-derived measurements. The acquisition parameters for all sequences are provided in Table 1.

**Table 1:** MR acquisition parameters for all sequences used in this study including T1 MPRAGE, T2 FLAIR, DTI, SWI, TOF, phase contrast (PC), and ASL.

Parameters	T1 MPRAGE	T2 FLAIR	DTI^a^	SWI	TOF	PC^b^	ASL^d^
Dimension	3D	3D	2D	3D	3D	2D	3D
Voxel size (mm^3^)	1 × 1 × 1	0.94 × 0.94 × 1	0.88 × 0.88 × 2	0.41 × 0.41 × 2	0.35 × 0.35 × 0.7	0.43 × 0.43 × 4	1.75 × 1.75 × 6.6
TR (ms)	7.25	8,000	6,000	37.10	24	6.33	4,735–5,197
TE (ms)	2.96	107.31	56.40	22.73	3.40	3.49	49.02
TI (ms)	400	2,161	–	–	–	–	2,025
Flip angle (°)	11	90	90	15	25	25	111
Matrix (pixels)	256 × 256	256 × 256	256 × 256	512 × 512	512 × 512	512 × 512	128 × 128
No. of slices	186	166	68	144	192	1-4^c^	36
No. of volumes	1	1	64	1	1	1–4 ^c^	2
FOV (mm)	256	240	224	210	180	220	224
Bandwidth (kHz)	244	390	1953	244	163	244	977

a Additional parameters for DTI include 64 gradient directions at b = 1,000 s/mm^2^.

b Additional parameters for PC include number of averages = 2; cardiac gating with beat rejection; number of temporal phases = 30–32 (dependent on heart rate; approximately 33 ms time-resolution); Velocity ENCoding value (VENC) = 120 cm/s (internal carotid and vertebral arteries).

c At minimum, one slice/volume is taken for coverage of the internal carotid and vertebral arteries. Time-permitting and anatomy-dependent, up to four slices/volumes are taken to fully characterize extracranial brain blood flow.

d Additional parameters for pseudo-continuous ASL include post label delay = 2025 ms, labeling duration = 1.8 s, number of excitations = 3, number of echos = 1, λ = 0.9 mL/g, $T_{1\text{blood}}$ = 1.65 s, and α = 0.85.

### 2.3 Phase contrast MRI sequence

**Figure 1:**
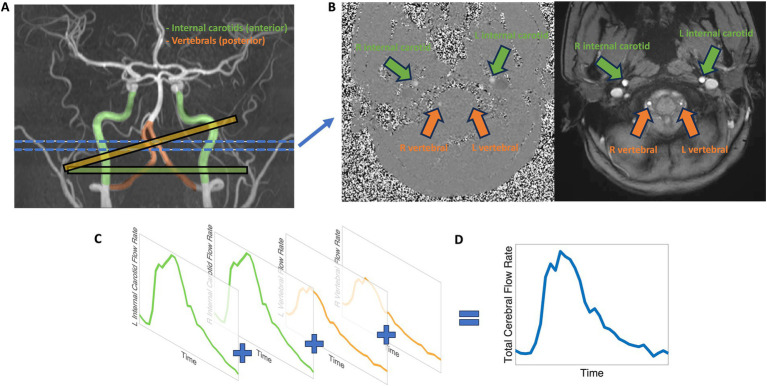
Visualization of internal carotid and vertebral artery flow measurements, representing anterior (green) and posterior (orange) cerebral blood flow, by phase contrast MRI. **(A)** The internal carotid arteries (green), representing the anterior circulation, and the vertebral arteries (orange), representing the posterior circulation are measured using phase contrast. Example 2D phase contrast plane perpendicular alignments are shown for the internal carotid arteries (green plane) and vertebral arteries (orange plane). The blue lines indicate the approximate target location for blood vessel measurement. **(B)** 2D phase contrast MRI produces cardiac-gated magnitude (right) and phase (left) cross-sectional slices. **(C)** Representation of typical internal carotid and vertebral blood flow profiles. **(D)** The sum of internal carotid and vertebral blood flow profiles characterizes extracranial total cerebral blood flow.

We quantified extracranial blood flow rates of the internal carotid and vertebral arteries from the 2D phase contrast sequence. Phase contrast is an MR velocimetry sequence that can be used to measure voxel-wise velocity maps of a 2D plane or 3D volume (Taylor and Draney, 2004). We used 2D phase contrast to measure cross-sectional blood flow of the primary arteries entering the brain (see Figure 1A), including the internal carotid and vertebral arteries that comprise the total blood flow into the brain (Zarrinkoob et al., 2015). A visualization of participant-specific blood vessel anatomy was first generated through the vascular angiography non-contrast TOF sequence, and a series of 2D phase contrast imaging planes were manually assigned to the internal carotid and vertebral vessels. To achieve maximum flow measurement accuracy, 2D phase contrast imaging planes were assigned perpendicular to the vessel of interest and away from rapid vessel curvature and bifurcation points with distal placement preferred to increase signal-to-noise ratio (Liu et al., 2014; Peng et al., 2015). In most participants, participant-specific complex arterial anatomy required that separate phase contrast planes be used to measure internal carotid and vertebral vessels with the number of planes ranging from one to four, depending on the complexity. Cases of vertebral artery aplasia were noted and verified by vascular angiography manual inspection. To achieve consistently at-rest flow and pulse wave measurements, the phase contrast sequence was assigned later in the overall MR sequence protocol. A Velocity ENCoding value (VENC) of 120 cm/s was sufficient to prevent any velocity aliasing in the vessels of interest, although at the expense of reduced signal to noise ratio for lower velocities (Nayak et al., 2015). Cardiac gating was acquired via pulse-oximeter measurements from the index finger; 30–32 temporal phases, representing a single averaged cardiac cycle, were acquired over the duration of the scan allowing for a time resolution of approximately 33 ms.

All blood vessel flow measurements were quantified with Medis QFlow v4.0 software (Burkhardt et al., 2023). A phase contrast scan consists of two components: a cardiac-gated anatomical cross-sectional plane and voxel-wise velocity map (see Figure 1B). Briefly, the post-processing procedure includes eddy current correction and semi-automatic vessel lumen contour detection after manual identification of the internal carotid and vertebral vessels, followed by extraction of velocity information within the contoured vessels with the calculation of average flow rate. The semi-automatic vessel lumen contour detection is performed on the first cardiac period, then is propagated, for each vessel, throughout the cardiac cycle to measure the time-dependent change in blood vessel area. For each cardiac phase, the velocity values contained within the blood vessel lumen are multiplied by the lumen area and summed to calculate out-of-plane flow rate. The average flow rate, in mL/min, is calculated as the mean cardiac period flow rate across the cardiac cycle for the blood vessel of interest (see Figure 1C). The sum of the internal carotid and vertebral artery average flow rates produces our measure of total cerebral blood flow (see Figure 1D).

To further segment extracranial flow patterns beyond total cerebral blood flow to the brain, driven by the large flow variance and regional supply differentiation of the carotid and vertebral blood vessels, we created multiple categories to describe extracranial circulations. ‘Anterior flow’ was defined as the sum of left and right internal carotid blood flow rates and represents the extracranial anterior-driven blood flow to the brain. ‘Posterior flow’ was defined as the sum of blood flow rates from both the left and right vertebral arteries, or from a single vertebral artery in cases of aplasia, representing the extracranial posterior-driven blood flow to the brain. ‘Total flow’ was the sum of anterior and posterior flow and represents the extracranial total blood flow to the brain.

All phase contrast data were filtered based on a quality assurance technique that includes manually checking for significant deviations from circular cross-sections of blood vessels and regularity of pulse waveform morphology. Quality issues were commonly due to participant motion, phase contrast plane misalignment (on a vessel-by-vessel basis), and/or irregular participant heart rate. The manual 2D plane alignment of the phase contrast MR sequence is difficult to accomplish across all blood vessels of interest due to complex and participant-dependent vascular anatomy (Liu et al., 2014). Due to MR protocol timing constraints, most often, two 2D phase contrast planes were acquired, with one plane aligned simultaneously to both internal carotid blood vessels and the second aligned to both vertebral blood vessels. Measuring flow rates within the internal carotid arteries was emphasized and, time-permitting, were reacquired if initial plane alignment was not optimal. Only phase contrast data that included at least one set of anterior and posterior circulation measurements that passed all stringent quality assurance checks were included in this study, which resulted in the collection of more anterior circulation phase contrast data than posterior circulation and total flow data.

### 2.4 MRI processing

Regional volumes and cortical thickness were quantified with FreeSurfer v6.0^1^ (Dale et al., 1999) with all gray matter, white matter, and ventricular segmentations verified by visual inspection. Left and right hemisphere regional volumes and cortical thickness were summed and averaged, respectively, for a bilateral quantification. Whole-brain WMH volume was quantified by fitting a single Gaussian curve to each FLAIR image intensity spectrum of the brain-extracted volume (Brickman et al., 2018). Then, brain voxels with intensity greater than the sigma threshold level of 2.1 standard deviations above the mean intensity value were labeled as WMH, summed, and multiplied by voxel dimensions to yield total volumes in cm^3^, and log transformed. All WMH volumes were verified by visual inspection with labeling corrected by manual editing if necessary. From the 64-direction DTI sequence, we estimated white matter microstructure integrity throughout white matter tracts with Tract-Based Spatial Statistics (TBSS, https://fsl.fmrib.ox.ac.uk/fsl/fslwiki/TBSS) (Smith et al., 2004; Smith et al., 2006). After brain registration to Montreal Neurological Institute (MNI) space, mean fractional anisotropy (FA) was calculated along a group-averaged FA skeleton of each major white matter tract. Left and right hemisphere white matter tracts were averaged for a composite mean value. Cerebral microbleed (MB) counts were derived semi-automatically, to improve identification efficiency, with the Microbleed Automated detection using Geometric Identification Criteria (MAGIC) toolbox applied to the SWI sequence (Chesebro et al., 2021). All labeled microbleed counts were verified by visual inspection and binarized to indicate the presence or absence of microbleeds. Briefly, the MAGIC toolbox uses spherical feature extraction algorithms to identify the hypointense effects of hemosiderin/iron deposits. All potential microbleeds were examined for mimics, such as calcium deposits and bone and blood vessel flow voids, by using an identification criterion that takes into account the location, intensity consistency and shape features of the hypointense effects (Greenberg et al., 2009). Pseudo-continuous ASL processing was performed following the standard approach recommended in the ASL consensus guidelines (Alsop et al., 2015). During sequence acquisition, the GE 3 T scanner performed on-scanner subtraction of control and label images to generate the perfusion-weighted image. Both the M0 image and perfusion-weighted image were then further processed using the consensus pseudo-continuous ASL cerebral blood flow quantification formula to calculate voxel-wise absolute cerebral blood flow values (Alsop et al., 2015). To ensure numerical stability, a threshold was applied to the M0 image to exclude voxels with low signal intensity, avoiding division by near-zero values. Following quantification, cerebral blood flow maps were registered to each participant’s T1-weighted anatomical image using bbregister in FreeSurfer, which implements boundary-based registration for high accuracy alignment. Cortical and subcortical masks derived from the Desikan-Killiany atlas were then applied to extract lobar and regional mean cerebral blood flow values. Finally, the sum of regional cerebral blood flow averages multiplied by regional brain volume and an assumed brain density estimated intracranially-derived total cerebral blood flow in mL/min (Su and Peng, 2024).

### 2.5 Statistical analysis

General linear models tested the association of total, anterior, and posterior blood flow rates with regional cortical thickness, regional volumes, total WMH volumes, white matter tract fractional anisotropy, and age. Separate models were run for each region in each imaging modality with only cortical thickness and regional volumes that are perfused by the supplying artery tested for associations (Liu et al., 2023). The Benjamini-Hochberg adjustment for multiple comparisons was applied to statistical analyses involving regional cortical thickness, regional volumes, and white matter tracts (Chen et al., 2017). Logit regression models tested the associations of total, anterior, and posterior blood flow rates with microbleed presence. All statistical models were adjusted for age, vascular risk factors, intracranial volume, and sex. A regression adjustment for intracranial volume was chosen over a proportional or normalization approach (Wang et al., 2024). The Bland–Altman method was used to compare total cerebral blood flow measurements obtained from phase contrast MRI and ASL (Giavarina, 2015). The Bland–Altman analysis included calculating the limits of agreement and testing for proportional bias between the two blood flow measurement techniques (Ludbrook, 2010). Statistical analysis was performed in RStudio 2023.03.1 with R version 4.2.1 and the additional packages of ‘performance’ and ‘naniar’ for analyses, and ‘ggplot2’, ‘ggforestplot’, ‘tidyverse’, ‘rempsyc’, and ‘blandr’ for visualization. All reported effect sizes and confidence intervals are standardized values. Logit model reporting includes odd ratios and *p*-values calculated from Pearson’s chi-squared test.

## 3 Results

Table 2 provides the demographic data for the 311 WHICAP study participants who received phase contrast MRI that passed the quality assurance checks. Due to quality assurance checks, the number of participants varied among total (*n* = 232), anterior (*n* = 292), and posterior (*n* = 241) blood flow measurements, but the demographic variables and differential missingness between each phase contrast subgroup were not different.

**Table 2:** Demographic data of study participants.

Characteristic	Study participants
	(*n* = 311)^a^
Age (years)	
Mean (SD)	76.6 (5.64)
Sex	
Men	97 (31.2%)
Women	214 (68.8%)
Education (years)	
Mean (SD)	12.8 (4.70)
Race and ethnicity	
Non-Hispanic White	75 (24.1%)
Non-Hispanic Black	73 (23.5%)
Hispanic or Latino/a/x	148 (47.6%)
Other	14 (4.5%)
Vascular risk factors	
Mean (SD)	1.27 (0.90)
Hypertension status	
No history of hypertension	83 (26.7%)
History of hypertension	228 (73.3%)
Diabetes status	
No history of diabetes	221 (71.1%)
History of diabetes	90 (28.9%)
Heart disease	
No history of heart disease	230 (74.0%)
History of heart disease	81 (26.0%)
Smoking	
No history of smoking	199 (64.0%)
Active and/or history of smoking	81 (26.0%)
BMI	
Mean (SD)	27.5 (3.83)

a Missing flow data from 79 (25%), 19 (6%), and 70 (23%) participants, respectively, from total, anterior, and posterior flow.

### 3.1 Association between extracranial blood flow and regional cortical thickness

**Figure 2:**
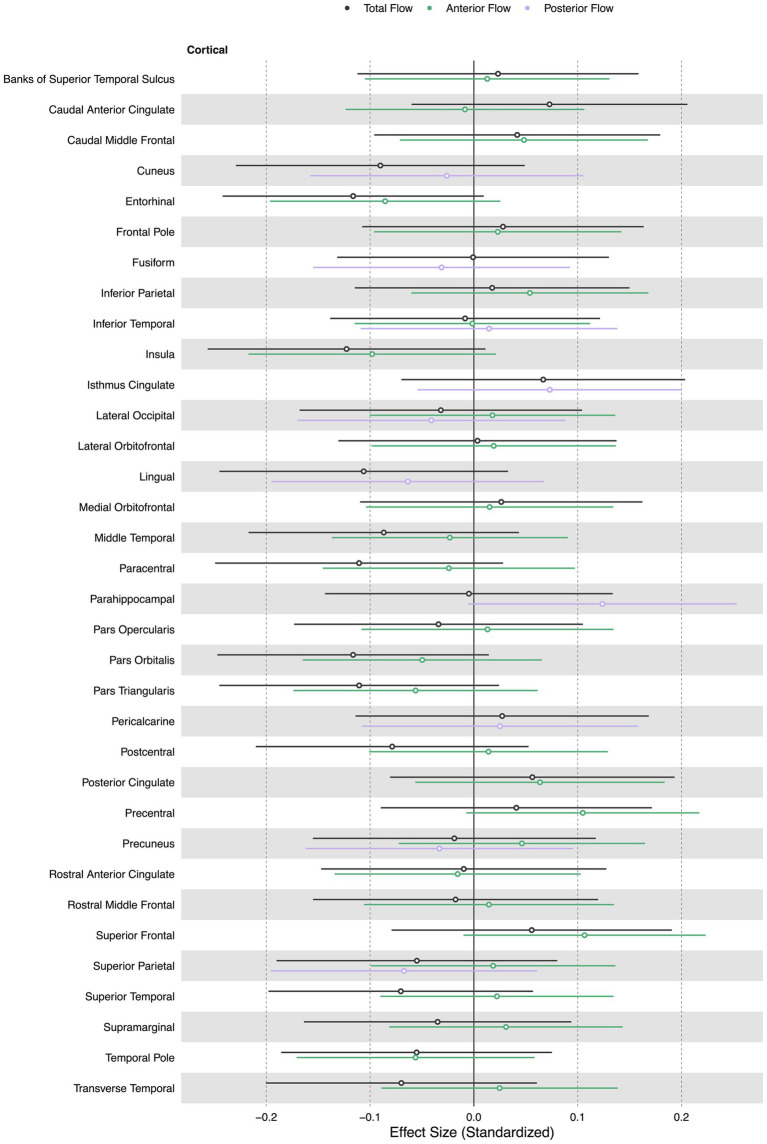
Summary forest plot showing the effect sizes of the relationship of extracranial total, anterior, and posterior cerebral blood flow with cortical thickness. Analyses are adjusted for age, vascular risk factors, intracranial volume, and sex, with corrections applied for multiple comparisons. Each bar represents the results of a separate linear model. Only regions that are perfused by the supplying artery are tested for associations (Liu et al., 2023). Filled and open circles represent significant and non-significant associations, respectively.

Total, anterior, and posterior cerebral blood flow were not associated with cortical thickness in any region. Figure 2 displays effect sizes of the relationship of extracranial total, anterior, and posterior cerebral blood flow with cortical thickness, with detailed statistical results provided in Supplementary materials section 2.

### 3.2 Association between extracranial blood flow and regional volume

Lower extracranial total cerebral blood flow was primarily associated with lower regional volumes in the caudal anterior cingulate (*β* = 0.19, *p* < 0.05), lateral orbitofrontal (*β* = 0.11, *p* < 0.05), precuneus (*β* = 0.17, *p* < 0.05), superior parietal (*β* = 0.13, *p* < 0.05), and pericalcarine cortices (*β* = 0.14, *p* < 0.05). Lower total cerebral blood flow was associated with larger third (*β* = − 0.20, *p* < 0.05), fourth (*β* = − 0.19, *p* < 0.05), and lateral ventricle volumes (*β* = − 0.13, *p* < 0.05).

Lower extracranial anterior cerebral blood flow was associated with lower regional volumes in the accumbens area region (*β* = 0.11, *p* < 0.05), caudal anterior cingulate (*β* = 0.13, *p* < 0.05), inferior parietal (*β* = 0.12, *p* < 0.05), lateral orbitofrontal (*β* = 0.10, *p* < 0.05), postcentral (*β* = 0.10, p < 0.05), posterior cingulate (*β* = 0.13, *p* < 0.05), precentral (*β* = 0.14, *p* < 0.05), rostral middle frontal (*β* = 0.13, *p* < 0.05), superior frontal (*β* = 0.10, *p* < 0.05), and precuneus cortices (*β* = 0.15, *p* < 0.05).

**Figure 3:**
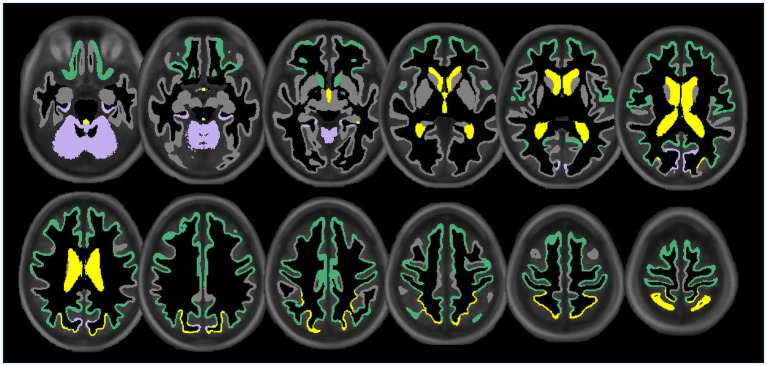
Illustration showing the significant associations of extracranial total (yellow), anterior (green), and posterior (purple) cerebral blood flow with cortical, subcortical, and ventricular system regional volume ROIs. Regional volume ROIs with nonsignificant associations with extracranial blood flow are shown in gray. Analyses are adjusted for age, vascular risk factors, intracranial volume, and sex, with corrections applied for multiple comparisons.

**Figure 4:**
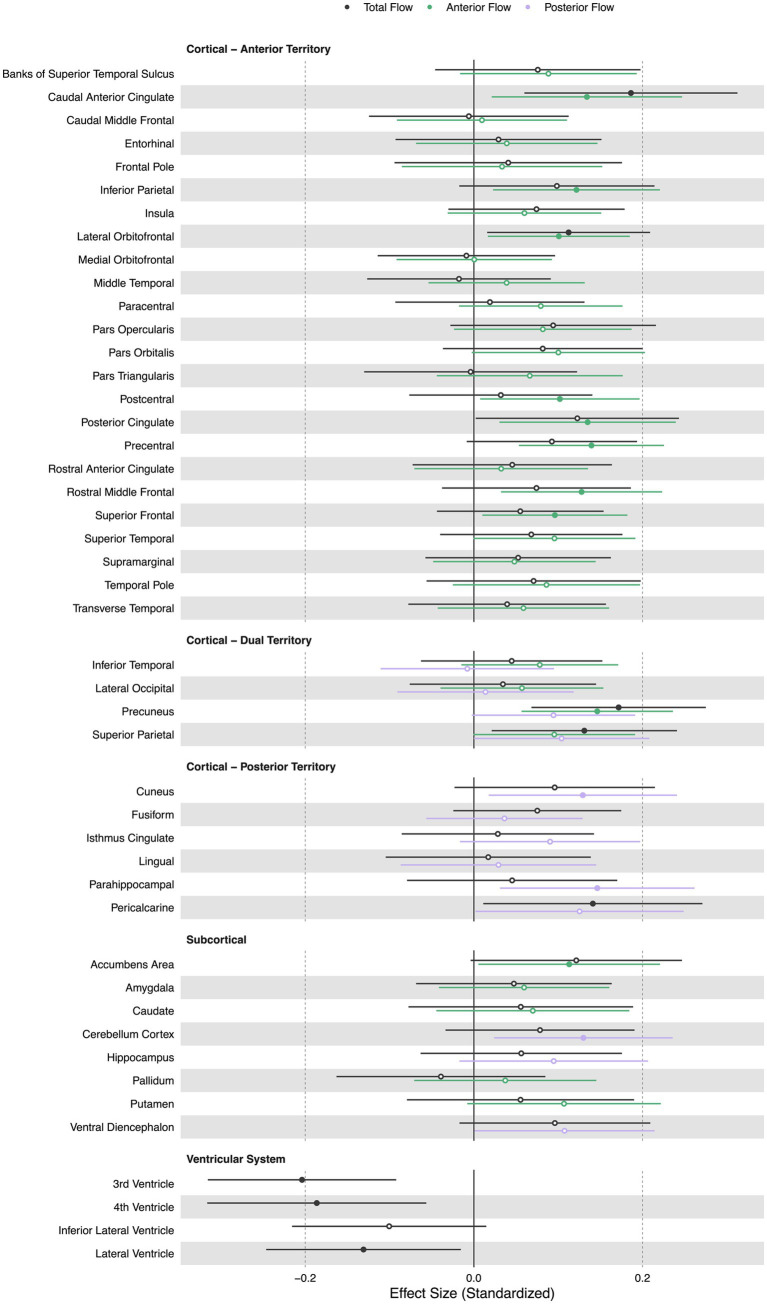
Summary forest plot showing the effect sizes of the relationship of extracranial total, anterior, and posterior cerebral blood flow with regional volumes. Analyses are adjusted for age, vascular risk factors, intracranial volume, and sex, with corrections applied for multiple comparisons. Each bar represents the results of a separate linear model. Organized by cortical, subcortical, and ventricular system regions. Only regions that are perfused by the supplying artery are tested for associations (Liu et al., 2023). Filled and open circles represent significant and non-significant associations, respectively.

Lower extracranial posterior cerebral blood flow was associated with lower regional volumes in the cuneus (*β* = 0.13, *p* < 0.05), parahippocampal (*β* = 0.15, *p* < 0.05), and cerebellum cortices (*β* = 0.13, *p* < 0.05). Figure 3 displays associations of extracranial total, anterior, and posterior cerebral blood flow with regional volumes. Figure 4 displays effect sizes of the relationship of extracranial total, anterior, and posterior cerebral blood flow with regional volumes, with detailed statistical results provided in Supplementary materials section 3.

### 3.3 Association between extracranial blood flow and white matter tract integrity

**Figure 5:**
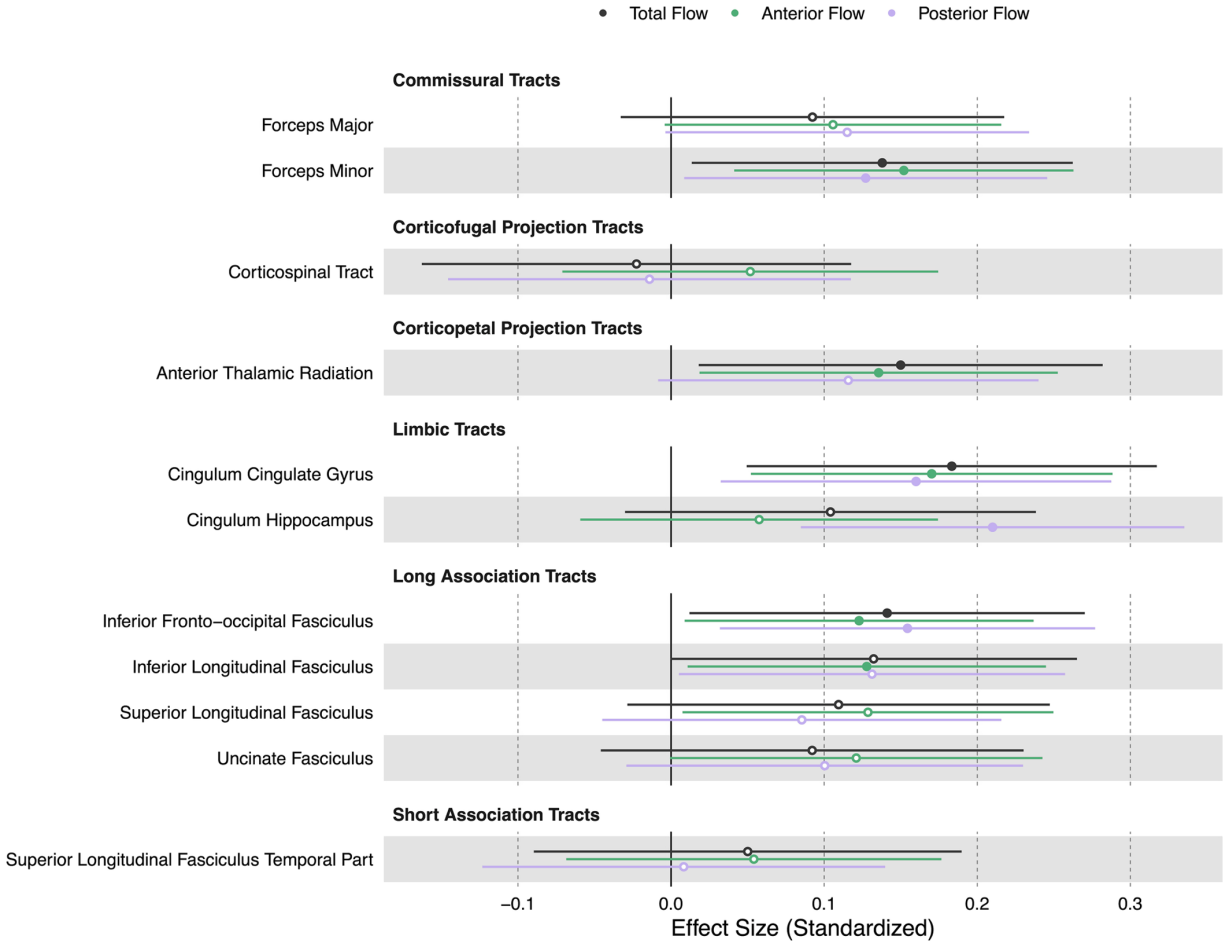
Summary forest plot showing the effect sizes of the relationship of extracranial total, anterior, and posterior cerebral blood flow with white matter tract integrity. Analyses are adjusted for age, vascular risk factors, intracranial volume, and sex, with corrections applied for multiple comparisons. Each bar represents the results of a separate linear model. Organized by commissural, projection, limbic, and association tracts. Filled and open circles represent significant and non-significant associations, respectively.

Lower extracranial total, anterior, and posterior cerebral blood flow were all associated with lower FA in white matter tracts including the forceps minor ($\beta_{\text{total}}$ = 0.14, $\beta_{\text{anterior}}$ = 0.15, $\beta_{\text{posterior}}$ = 0.13, *p* < 0.05), cingulum cingulate gyrus ($\beta_{\text{total}}$ = 0.18, $\beta_{\text{anterior}}$ = 0.17, $\beta_{\text{posterior}}$ = 0.16, p < 0.05), and inferior fronto-occipital fasciculus ($\beta_{\text{total}}$ = 0.14, $\beta_{\text{anterior}}$ = 0.12, $\beta_{\text{posterior}}$ = 0.15, *p* < 0.05). Additionally, lower total cerebral blood flow was associated with lower FA in the anterior thalamic radiation (*β* = 0.15, *p* < 0.05). Lower anterior cerebral blood flow was associated with lower FA in the anterior thalamic radiation (*β* = 0.14, *p* < 0.05) and inferior longitudinal fasciculus (*β* = 0.13, *p* < 0.05). Extracranial posterior cerebral blood flow was associated with lower FA in the cingulum hippocampus (*β* = 0.21, *p* < 0.05). Figure 5 displays effect sizes of the relationship of extracranial total, anterior, and posterior cerebral blood flow with white matter tract integrity, with detailed statistical results provided in Supplementary materials section 4.

### 3.4 Association between extracranial blood flow and WMH volume

**Figure 6:**
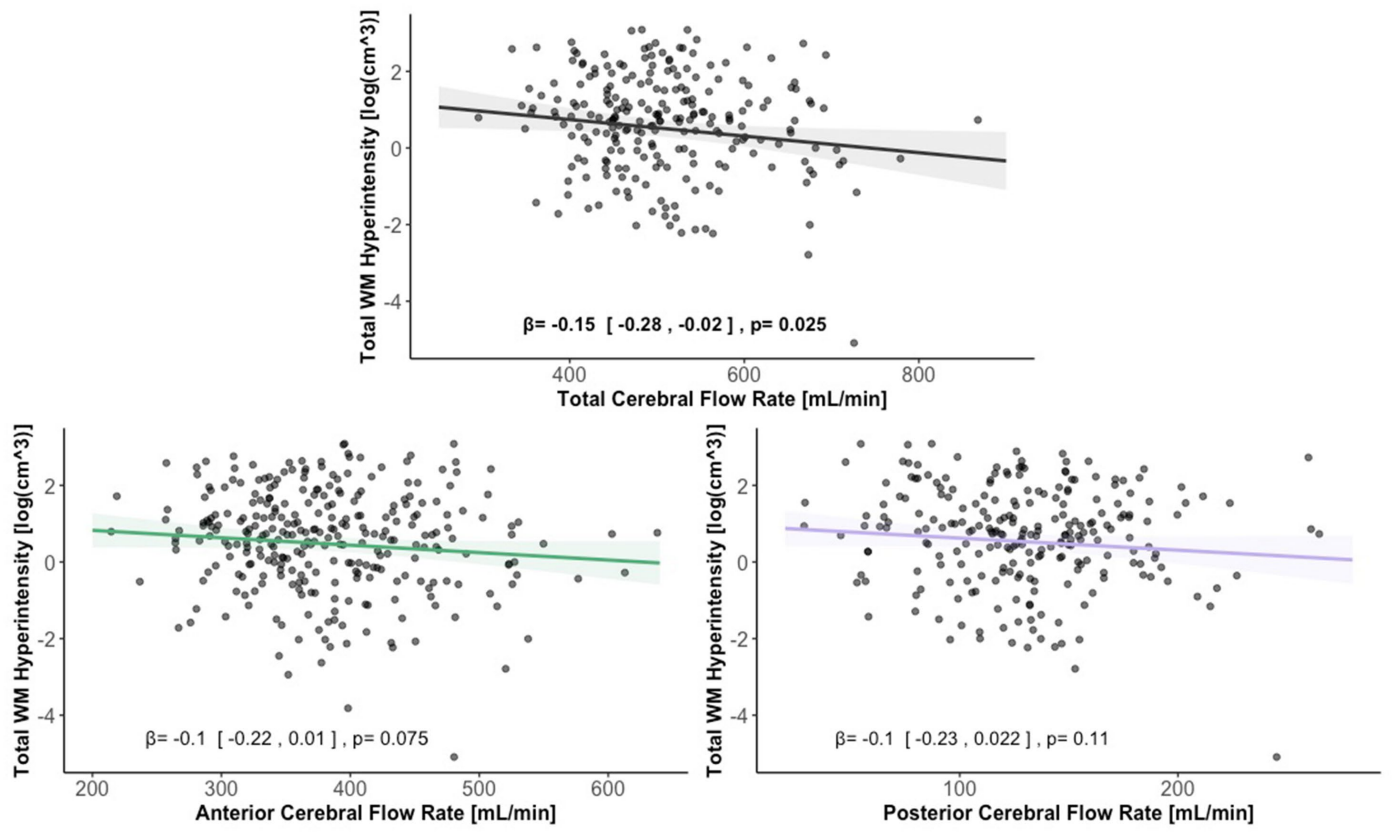
Scatter plots displaying the association between extracranial total, anterior, and posterior cerebral blood flow and total WMH volume. Linear regression curves, with 95% confidence intervals, are adjusted for age, vascular risk factors, intracranial volume, and sex. Scatter plot datapoints are visualized on unadjusted values.

Lower total extracranial cerebral blood flow was associated with greater total WMH volume [$\beta_{\text{total}}$ = − 0.15, 95% CI (−0.28, −0.02), *p* < 0.05]. Anterior and posterior cerebral blood flow were not associated with total WMH volume [$\beta_{\text{anterior}}$ = − 0.10, 95% CI (−0.22, 0.01), *p* > 0.05, $\beta_{\text{posterior}}$ = − 0.10, 95% CI (−0.23, 0.02), *p* > 0.05]. Figure 6 displays scatter plots with the association of extracranial total, anterior, and posterior cerebral blood flow with total WMH volume.

### 3.5 Association of extracranial blood flow and cerebral microbleeds

Total, anterior, and posterior cerebral blood flow were not associated with presence of cerebral microbleeds [$\beta_{\text{total}}$ = 0.19, 95% CI (−0.18, 0.57), OR = 1.00, *p* > 0.05, $\beta_{\text{anterior}}$ = 0.16, 95% CI (−0.15, 0.48), OR = 1.00, *p* > 0.05, $\beta_{\text{posterior}}$ = 0.089, 95% CI (−0.27, 0.45), OR = 1.00, *p* > 0.05].

### 3.6 Association of age with blood flow

Older age was associated with lower total and anterior extracranial cerebral blood flow [$\beta_{\text{total}}$ = − 0.13, 95% CI (−0.26, −0.012), *p* < 0.05, $\beta_{\text{anterior}}$ = − 0.17, 95% CI (−0.28, −0.06), p < 0.05]. Age was not associated with posterior extracranial cerebral blood flow [$\beta_{\text{posterior}}$ = − 0.017, 95% CI (−0.14, 0.11), *p* > 0.05].

### 3.7 Association of extracranial blood flow with intracranial blood flow measurements

Extracranially-derived phase contrast measurement of total cerebral blood flow was associated with intracranially-derived ASL total cerebral blood flow [*β* = 0.62, 95% CI (0.51, 0.72), *p* < 0.05]. A Bland–Altman analysis shows a proportional bias between phase contrast and ASL-derived total blood flow measures [*β* = 0.15, 95% CI (0.02, 0.28), *p* < 0.05]. The mean bias between methods was 268.78 [72.2% of mean values, 95% CI (259.13, 278.44)] ± standard deviation of 75.32 (20.2% of mean values) mL/min. The 95% limits of agreement ranged from 124.13 to 413.45 mL/min [38.9% of mean values, 95% $CI_{\text{lower}}$ (107.61, 140.65), 95% $CI_{\text{upper}}$ (396.93, 429.97)]. Supplementary Figures S1 and S2, provided in Supplementary materials section 1, visualize the total cerebral blood flow measurement associations and Bland–Altman analysis, respectively. All intracranially-derived ASL total cerebral blood flow associations with regional cortical thickness, regional volume, white matter tract integrity, WMH volume, and age, for comparison with extracranially-derived phase contrast measurements, are provided with detailed statistics in both figures and tables in Supplementary materials sections 2–6. Intracranially-derived ASL total cerebral blood flow was not associated with presence of cerebral microbleeds [$\beta_{\text{total}-\mathrm{ASL}}$ = 0.89, 95% CI (−0.35, 0.3), OR = 1.00, *p* > 0.05]. In summary, there is a systematic proportional bias with 38.9% of mean value limit of agreement between measurements of intracranially-derived pseudo-continuous ASL and extracranially-derived phase contrast total cerebral blood flow, indicating that the subsequent associations between blood flow and markers of neurodegeneration may not be comparable between methodologies.

## 4 Discussion

We found that lower extracranial cerebral blood flow is associated with lower cortical regional volumes, lower white matter tract integrity, and higher WMH volume in older adults. While we cannot infer causality, our findings are consistent with the guiding conceptual framework that decreased extracranial blood flow may be one important factor that contributes to neurodegeneration and vascular brain injury in older adults.

The regional volume correlates of extracranial blood flow may identify gray matter regions of the brain that are particularly vulnerable to alterations in blood flow supply. Further investigation into the microvascular beds of these regions could provide evidence of relatively vulnerable vascular structure and function (Moody et al., 1990). Additionally, the regional associations we observed could partially explain reported regional cortical atrophy in aging (Sele et al., 2020; Thambisetty et al., 2010), and may be related to age-related cerebrovascular network remodeling (Bennett et al., 2024). The association between ventricular volume and extracranial blood flow provides convergent evidence that lower global blood supply may contribute to brain atrophy (Appelman et al., 2008; Ma et al., 2025), with the cumulative effect of atrophy being represented in higher ventricular volume (Madsen et al., 2015).

The white matter tracts associated with extracranial blood flow were in regions that are potentially susceptible to hypoperfusion in older adults. Notably, we observed associations between extracranial cerebral blood flow and many of the white matter tracts we tested, suggesting that extracranial blood flow may have a whole-brain impact on white matter microstructure. In particular, deep cerebral white matter is last-in-line to be perfused in the brain and can have single-source arterial supply with high variability of vascularization (Smirnov et al., 2021), potentially making it vulnerable to disruptions in perfusion. As white matter tracts transcend cerebral arterial territories, and thus have the potential to be influenced by changes of blood flow at numerous points along the various perfusion pathways, phase contrast MR may be a uniquely suited methodology to investigate the link between extracranial blood flow supply and white matter tract integrity. Our findings support the study hypothesis that extracranial blood flow in older age is coupled with brain-wide white matter microstructure. We suggest that chronic hypoperfusion may be one of the mechanisms contributing to white matter disruption, though causal studies are required to confirm this possibility. Age-related decreases in fractional anisotropy consistently occur in a majority of the white matter tracts across the whole brain including association, commissural, limbic, and sensorimotor tracts (de Groot et al., 2015; Pini et al., 2016), an effect that could be partially explained by extracranial cerebral blood flow. Our findings on the relationship between CBF and white matter microstructure align with studies of normal aging adults. In ASL-measured CBF and DTI-measured white matter microstructure studies, lower cortical and subcortical CBF correlated with lower fractional anisotropy (Chen et al., 2013; Bouhrara et al., 2022) and lower subcortical myelin water fraction (Bouhrara et al., 2020). These associations were observed at both whole-brain and lobar levels, even after statistically controlling for age (Chen et al., 2013; Bouhrara et al., 2022; Bouhrara et al., 2020) and vascular risk factors (Chen et al., 2013; Bouhrara et al., 2020). Moreover, a longitudinal study of middle-aged adults found that over 5 years, lower cortical CBF predicted greater decline in fractional anisotropy across whole-brain and nearly all lobar white matter regions (Bouhrara et al., 2022). Collectively, these findings reinforce the hypothesis that white matter microstructure, including myelin homeostasis and tissue integrity, is particularly vulnerable to hypoperfusion (Chen et al., 2013; Bouhrara et al., 2022; Bouhrara et al., 2020).

We found an association of lower total extracranial cerebral blood flow with higher WMH volume. Our findings are consistent with other studies (Bisschops et al., 2004; Han et al., 2022; Vernooij et al., 2008), though pulsatile-based phase contrast measures may be more strongly associated with (Pahlavian et al., 2021). White matter hyperintensities in the context of aging are indicative of small vessel cerebrovascular disease and are caused by, among other factors, hypoperfusion (Laing et al., 2020). Additionally, regions of WMH are associated with lower localized cerebral blood flow (Bisschops et al., 2004; Brickman et al., 2009; Promjunyakul et al., 2015) and the degree of cerebral blood flow decline is associated with WMH progression (Han et al., 2022). While our findings only demonstrate an association between extracranial cerebral blood flow and WMH volume, prior studies using longitudinal and mechanistic evidence support a potential causal link, suggesting that both global and regional cerebral hypoperfusion may promote or exacerbate small vessel cerebrovascular disease.

Our observed relationship between age and blood flow is consistent with previous reports. Previous reports observe decreases in total cerebral blood flow, measured with phase contrast MRI, with increasing age (Zarrinkoob et al., 2015; Zhao et al., 2007; Amin-Hanjani et al., 2015; Tarumi et al., 2014; Buijs et al., 1998), driven by both anterior and posterior circulations (Zarrinkoob et al., 2015; Zhao et al., 2007; Amin-Hanjani et al., 2015). While we did not observe age-related decreases in the posterior circulation, the age-related total cerebral blood flow difference was about −2.28 mL/min per year, driven by the anterior blood flow, and is similar to the rate of decrease found in other studies (Zarrinkoob et al., 2015; Zhao et al., 2007; Amin-Hanjani et al., 2015; Tarumi et al., 2014; Buijs et al., 1998).

This study has some limitations to be considered. The accuracy and reliability of blood flow quantification using phase contrast MRI has been questioned in previous research (Dolui et al., 2016). However, our protocol incorporated recommended quality assessment steps to uphold high standards for accuracy of flow measurement (Peng et al., 2015; Wentland et al., 2013), but these steps led to unequal numbers of participants with total, anterior, and posterior cerebral blood flow measurements and thus statistical comparisons are not equivalent across flow type. Alignment of a dedicated 2D phase contrast plane for each blood vessel individually could improve participant inclusion. Further, a phase contrast velocity encoding value of 120 cm/s was sufficient to prevent any phase aliasing but came at the expense of signal to noise ratio for measurement of lower velocities. Another study limitation is the cross-sectional design, which limits our ability to infer causality and temporality. While a causal framework guided the research design, we cannot exclude the possibility of reverse causality or bidirectional relationships between extracranial cerebral blood flow and markers of neurodegeneration and cerebrovascular injury (Ma et al., 2025; Zonneveld et al., 2015).

In summary, we observed that lower extracranial cerebral blood flow, quantified by total, anterior, and posterior circulations, is associated with lower white matter tract integrity in multiple tracts, including the forceps minor, cingulum cingulate gyrus, and inferior fronto-occipital fasciculus. Additionally, each flow type exhibits distinct tract-specific associations. Lower total extracranial cerebral blood flow is associated with higher WMH volume, a marker of small vessel cerebrovascular disease. Lower total and anterior extracranial flow are associated with lower regional volumes in the caudal anterior cingulate, lateral orbitofrontal, and precuneus cortices, with all flow types showing additional region-specific volumetric associations. Older age is associated with lower total and anterior extracranial cerebral blood flow.

The measurement of extracranial cerebral blood flow, through phase contrast MRI, is particularly useful in the investigation of wide-spanning indices of brain health such as white matter tract integrity and WMH. Additionally, consideration of extracranial brain perfusion is necessary to fully understand and characterize local blood flow and its neurobiological and behavioral correlates. The bridge between extracranial and local blood flow is influenced by individual-, aging- and pathology-specific interdependent vascular factors, including cerebral blood vessel network remodeling (Fisher et al., 2022; Thore et al., 2007; Bia et al., 2011), cerebral perfusion pressure (Meng et al., 2015), dynamic cerebrovascular blood vessel behavior (Stefanidis et al., 2019), and neurovascular coupling (Shabir et al., 2018). Our findings of a proportional bias between extracranially-derived and intracranially-derived measures of total cerebral blood flow indicate that pseudo-continuous ASL measurements may not be a reliable proxy for phase contrast measurements of total cerebral blood flow. Separate measurements of intracranially-derived regional flow and extracranially-derived total flow may be a complementary configuration of techniques to characterize blood flow of the brain. Further investigation into the relationship among white matter damage, such as white matter tracts and WMH, extracranial cerebral blood flow, regional perfusion, and cognition is needed in the context of aging and neurodegenerative diseases. In particular, regions of increased vulnerability to alterations in cerebral blood flow, such as regions of deep white matter, are a target to be investigated further.

## Data Availability

The datasets presented in this article are not readily available because data will only be available to qualified investigators with approval from study investigators. Requests to access the datasets should be directed to AMB, amb2139@columbia.edu.

## Footnotes

^1^ https://surfer.nmr.mgh.harvard.edu/

## References

[ref1] Alsop D. C. Detre J. A. Golay X. Günther M. Hendrikse J. Hernandez-Garcia L.. (2015). Recommended implementation of arterial spin-labeled perfusion MRI for clinical applications: a consensus of the ISMRM perfusion study group and the European consortium for ASL in dementia. Magn. Reson. Med. 73, 102–116. doi: 10.1002/mrm.25197, PMID: 24715426 PMC4190138

[ref2] Amin-Hanjani S. Du X. J. Pandey D. K. Thulborn K. R. Charbel F. T. (2015). Effect of age and vascular anatomy on blood flow in major cerebral vessels. J. Cereb. Blood Flow Metab. 35, 312–318. doi: 10.1038/jcbfm.2014.203, PMID: 25388677 PMC4426749

[ref3] Appelman A. P. A. Van der Graaf Y. Vincken K. L. Tiehuis A. M. Witkamp T. D. Mali W. P. T. M.. (2008). Total cerebral blood flow, white matter lesions and brain atrophy: the SMART-MR study. J. Cerebr. Blood Flow. Metab. 28, 633–639. doi: 10.1038/sj.jcbfm.9600563, PMID: 17912270

[ref4] Aslan S. Xu F. Wang P. L. Uh J. Yezhuvath U. S. van Osch M.. (2010). Estimation of labeling efficiency in pseudocontinuous arterial spin labeling. Magn. Reson. Med. 63, 765–771. doi: 10.1002/mrm.22245, PMID: 20187183 PMC2922009

[ref5] Avila J. F. Renteria M. A. Jones R. N. Vonk J. M. J. Turney I. Sol K.. (2021). Education differentially contributes to cognitive reserve across racial/ethnic groups. Alzheimers Dement. 17, 70–80. doi: 10.1002/alz.12176, PMID: 32827354 PMC8376080

[ref6] Bahrani A. A. Powell D. K. Yu G. Q. Johnson E. S. Jicha G. A. Smith C. D. (2017). White matter hyperintensity associations with cerebral blood flow in elderly subjects stratified by cerebrovascular risk. J. Stroke Cerebrovasc. Dis. 26, 779–786. doi: 10.1016/j.jstrokecerebrovasdis.2016.10.017, PMID: 28063772 PMC5473621

[ref7] Bastos-Leite A. J. Kuijer J. P. A. Rombouts S. A. R. B. Sanz-Arigita E. van Straaten E. C. Gouw A. A.. (2008). Cerebral blood flow by using pulsed arterial spin-labeling in elderly subjects with white matter hyperintensities. Am. J. Neuroradiol. 29, 1296–1301. doi: 10.3174/ajnr.A1091, PMID: 18451090 PMC8119130

[ref8] Bennett H. C. Zhang Q. Wu Y. T. Manjila S. B. Chon U. Shin D.. (2024). Aging drives cerebrovascular network remodeling and functional changes in the mouse brain. Nat. Commun. 15:6398. doi: 10.1038/s41467-024-50559-8, PMID: 39080289 PMC11289283

[ref9] Bia D. Zocalo Y. Farro I. Torrado J. Farro F. Florio L.. (2011). Integrated evaluation of age-related changes in structural and functional vascular parameters used to assess arterial aging, subclinical atherosclerosis, and cardiovascular risk in Uruguayan adults: CUiiDARTE project. Int. J. Hypertens. 2011:587303, 1–12. doi: 10.4061/2011/587303, PMID: 22187622 PMC3235479

[ref10] Bisschops R. H. C. van der Graaf Y. Mali W. P. T. M. van der Grond J. grp Ss (2004). High total cerebral blood flow is associated with a decrease of white matter lesions. J. Neurol. 251, 1481–1485. doi: 10.1007/s00415-004-0569-y, PMID: 15645347

[ref11] Bouhrara M. Alisch J. S. R. Khattar N. Kim R. W. Rejimon A. C. Cortina L. E.. (2020). Association of cerebral blood flow with myelin content in cognitively unimpaired adults. BMJ Neurol. Open 2:e000053. doi: 10.1136/bmjno-2020-000053, PMID: 33681786 PMC7903181

[ref12] Bouhrara M. Triebswetter C. Kiely M. Bilgel M. Dolui S. Erus G.. (2022). Association of Cerebral Blood Flow with Longitudinal Changes in cerebral microstructural integrity in the coronary artery risk development in young adults (CARDIA) study. JAMA Netw. Open 5:e2231189. doi: 10.1001/jamanetworkopen.2022.31189, PMID: 36094503 PMC9468885

[ref13] Brickman A. M. Manly J. J. Honig L. S. Sanchez D. Reyes-Dumeyer D. Lantigua R. A.. (2021). Plasma p-tau181, p-tau217, and other blood-based Alzheimer's disease biomarkers in a multi-ethnic, community study. Alzheimers Dement. 17, 1353–1364. doi: 10.1002/alz.12301, PMID: 33580742 PMC8451860

[ref14] Brickman A. M. Tosto G. Gutierrez J. Andrews H. Gu Y. Narkhede A.. (2018). An MRI measure of degenerative and cerebrovascular pathology in Alzheimer disease. Neurology 91, E1402–e1412. doi: 10.1212/WNL.0000000000006310, PMID: 30217936 PMC6177275

[ref15] Brickman A. M. Zahra A. Muraskin J. Steffener J. Holland C. M. Habeck C.. (2009). Reduction in cerebral blood flow in areas appearing as white matter hyperintensities on magnetic resonance imaging. Psychiatry Res. Neuroimaging 172, 117–120. doi: 10.1016/j.pscychresns.2008.11.006, PMID: 19324534 PMC2763417

[ref16] Buijs P. C. Krabbe-Hartkamp M. J. Bakker C. J. G. de Lange E. E. Ramos L. M. P. Breteler M. M. B.. (1998). Effect of age on cerebral blood flow: measurement with ungated two-dimensional phase-contrast MR angiography in 250 adults. Radiology 209, 667–674. doi: 10.1148/radiology.209.3.9844657 9844657

[ref17] Burkhardt B. E. U. Kellenberger C. J. Callaghan F. M. Valsangiacomo Buechel E. R. Geiger J. (2023). Flow evaluation software for four-dimensional flow MRI: a reliability and validation study. Radiol. Med. 128, 1225–1235. doi: 10.1007/s11547-023-01697-4, PMID: 37620674 PMC10547653

[ref18] Caunca M. R. De Leon-Benedetti A. Latour L. Leigh R. Wright C. B. (2019). Neuroimaging of cerebral small vessel disease and age-related cognitive changes. Front. Aging Neurosci. 11:11. doi: 10.3389/fnagi.2019.00145, PMID: 31316367 PMC6610261

[ref19] Chen S. Y. Feng Z. Yi X. L. (2017). A general introduction to adjustment for multiple comparisons. J. Thorac. Dis. 9, 1725–1729. doi: 10.21037/jtd.2017.05.34, PMID: 28740688 PMC5506159

[ref20] Chen J. J. Rosas H. D. Salat D. H. (2013). The relationship between cortical blood flow and sub-cortical white-matter health across the adult age span. PLoS One 8:e56733. doi: 10.1371/journal.pone.0056733, PMID: 23437228 PMC3578934

[ref21] Chesebro A. G. Amarante E. Lao P. J. Meier I. B. Mayeux R. Brickman A. M. (2021). Automated detection of cerebral microbleeds on T2*-weighted MRI. Sci. Rep. 11:4004. doi: 10.1038/s41598-021-83607-0, PMID: 33597663 PMC7889861

[ref22] Dale A. M. Fischl B. Sereno M. I. (1999). Cortical surface-based analysis. I. Segmentation and surface reconstruction. NeuroImage 9, 179–194. doi: 10.1006/nimg.1998.0395, PMID: 9931268

[ref23] de Groot M. Ikram M. A. Akoudad S. Krestin G. P. Hofman A. van der Lugt A.. (2015). Tract-specific white matter degeneration in aging: the Rotterdam study. Alzheimers Dement. 11, 321–330. doi: 10.1016/j.jalz.2014.06.011, PMID: 25217294

[ref24] Dolui S. Wang Z. Wang D. J. J. Mattay R. Finkel M. Elliott M.. (2016). Comparison of non-invasive MRI measurements of cerebral blood flow in a large multisite cohort. J. Cereb. Blood Flow Metab. 36, 1244–1256. doi: 10.1177/0271678X16646124, PMID: 27142868 PMC4929707

[ref25] Fisher R. A. Miners J. S. Love S. (2022). Pathological changes within the cerebral vasculature in Alzheimer's disease: new perspectives. Brain Pathol. 32:e13061. doi: 10.1111/bpa.13061, PMID: 35289012 PMC9616094

[ref26] Gelfand B. D. Epstein F. H. Blackman B. R. (2006). Spatial and spectral heterogeneity of time-varying shear stress profiles in the carotid bifurcation by phase-contrast MRI. J. Magn. Reson. Imaging 24, 1386–1392. doi: 10.1002/jmri.20765, PMID: 17083089

[ref27] Giavarina D. (2015). Understanding bland Altman analysis. Biochem. Med. 25, 141–151. doi: 10.11613/BM.2015.015, PMID: 26110027 PMC4470095

[ref28] Ginsberg M. D. (2016). Expanding the concept of neuroprotection for acute ischemic stroke: the pivotal roles of reperfusion and the collateral circulation. Prog. Neurobiol. 145-146, 46–77. doi: 10.1016/j.pneurobio.2016.09.002, PMID: 27637159

[ref29] Greenberg S. M. Vernooij M. W. Cordonnier C. Viswanathan A. Al-Shahi Salman R. Warach S.. (2009). Cerebral microbleeds: a guide to detection and interpretation. Lancet Neurol. 8, 165–174. doi: 10.1016/S1474-4422(09)70013-4, PMID: 19161908 PMC3414436

[ref30] Han H. Lin Z. Soldan A. Pettigrew C. Betz J. F. Oishi K.. (2022). Longitudinal changes in global cerebral blood flow in cognitively Normal older adults: a phase-contrast MRI study. J. Magn. Reson. Imaging 56, 1538–1545. doi: 10.1002/jmri.28133, PMID: 35218111 PMC9411265

[ref31] Jung Y. Wong E. C. Liu T. T. (2010). Multiphase Pseudocontinuous arterial spin labeling (MP-PCASL) for robust quantification of cerebral blood flow. Magn. Reson. Med. 64, 799–810. doi: 10.1002/mrm.22465, PMID: 20578056

[ref32] Koerte I. Haberl C. Schmidt M. Pomschar A. Lee S. Rapp P.. (2013). Inter- and intra-rater reliability of blood and cerebrospinal fluid flow quantification by phase-contrast MRI. J. Magn. Reson. Imaging 38, 655–662. doi: 10.1002/jmri.24013, PMID: 23371821 PMC3644311

[ref33] Laing K. K. Simoes S. Baena-Caldas G. P. Lao P. J. Kothiya M. Igwe K. C.. (2020). Cerebrovascular disease promotes tau pathology in Alzheimer's disease. Brain Commun. 2:fcaa132. doi: 10.1093/braincomms/fcaa132, PMID: 33215083 PMC7660042

[ref34] Liu C. F. Hsu J. Xu X. Kim G. Sheppard S. M. Meier E. L.. (2023). Digital 3D brain MRI arterial territories atlas. Sci. Data 10:74. doi: 10.1038/s41597-022-01923-0, PMID: 36739282 PMC9899211

[ref35] Liu P. Y. Lu H. Z. Filbey F. M. Pinkham A. E. McAdams C. J. Adinoff B.. (2014). Automatic and reproducible positioning of phase-contrast MRI for the quantification of global cerebral blood flow. PLoS One 9:e95721. doi: 10.1371/journal.pone.0095721, PMID: 24787742 PMC4008483

[ref36] Luchsinger J. A. Reitz C. Honig L. S. Tang M. X. Shea S. Mayeux R. (2005). Aggregation of vascular risk factors and risk of incident Alzheimer disease. Neurology 65, 545–551. doi: 10.1212/01.wnl.0000172914.08967.dc, PMID: 16116114 PMC1619350

[ref37] Ludbrook J. (2010). Confidence in Altman-bland plots: a critical review of the method of differences. Clin. Exp. Pharmacol. Physiol. 37, 143–149. doi: 10.1111/j.1440-1681.2009.05288.x, PMID: 19719745

[ref38] Ma Y. Bos D. Wolters F. J. Niessen W. Hofman A. Ikram M. A.. (2025). Changes in cerebral hemodynamics and progression of subclinical vascular brain disease: a population-based cohort study. Stroke 56, 95–104. doi: 10.1161/STROKEAHA.124.047593, PMID: 39633567 PMC11661930

[ref39] MacDonald M. E. Pike G. B. (2021). MRI of healthy brain aging: a review. NMR Biomed. 34:e4564. doi: 10.1002/nbm.4564, PMID: 34096114

[ref40] Madsen S. K. Gutman B. A. Joshi S. H. Toga A. W. Jack C. R. Weiner M. W.. (2015). Mapping ventricular expansion onto cortical gray matter in older adults. Neurobiol. Aging 36, S32–S41. doi: 10.1016/j.neurobiolaging.2014.03.044 25311280 PMC4268107

[ref41] Meng L. Z. Hou W. G. Chui J. Han R. Q. Gelb A. W. (2015). Cardiac output and cerebral blood flow. Anesthesiology 123, 1198–1208. doi: 10.1097/ALN.0000000000000872, PMID: 26402848

[ref42] Moody D. M. Bell M. A. Challa V. R. (1990). Features of the cerebral vascular pattern that predict vulnerability to perfusion or oxygenation deficiency - an anatomic study. Am. J. Neuroradiol. 11, 431–439. 2112304 PMC8367475

[ref43] Mynard J. P. Steinman D. A. (2013). Effect of velocity profile skewing on blood velocity and volume flow waveforms derived from maximum Doppler spectral velocity. Ultrasound Med. Biol. 39, 870–881. doi: 10.1016/j.ultrasmedbio.2012.11.006, PMID: 23453373

[ref44] Nayak K. S. Nielsen J. F. Bernstein M. A. Markl M. Gatehouse P. D. Botnar R. M.. (2015). Cardiovascular magnetic resonance phase contrast imaging. J. Cardiovasc. Magn. Reson. 17:71. doi: 10.1186/s12968-015-0172-7, PMID: 26254979 PMC4529988

[ref45] Ozpar R. Dinc Y. Nas O. F. Inecikli M. F. Parlak M. Hakyemez B. (2023). Arterial transit artifacts observed on arterial spin labeling perfusion imaging of carotid artery stenosis patients: what are counterparts on symptomatology, dynamic susceptibility contrast perfusion, and digital subtraction angiography? J. Neuroradiol. 50, 407–414. doi: 10.1016/j.neurad.2022.08.005, PMID: 36067966

[ref46] Pahlavian S. H. Wang X. Ma S. Zheng H. Casey M. D'Orazio L. M.. (2021). Cerebroarterial pulsatility and resistivity indices are associated with cognitive impairment and white matter hyperintensity in elderly subjects: a phase-contrast MRI study. J. Cereb. Blood Flow Metab. 41, 670–683. doi: 10.1177/0271678X20927101 32501154 PMC7922759

[ref47] Peng S. L. Su P. Wang F. N. Cao Y. Zhang R. Lu H. Z.. (2015). Optimization of phase-contrast MRI for the quantification of whole-brain cerebral blood flow. J. Magn. Reson. Imaging 42, 1126–1133. doi: 10.1002/jmri.24866, PMID: 25676350 PMC4532651

[ref48] Pini L. Pievani M. Bocchetta M. Altomare D. Bosco P. Cavedo E.. (2016). Brain atrophy in Alzheimer's disease and aging. Ageing Res. Rev. 30, 25–48. doi: 10.1016/j.arr.2016.01.002, PMID: 26827786

[ref49] Promjunyakul N. Lahna D. Kaye J. A. Dodge H. H. Erten-Lyons D. Rooney W. D.. (2015). Characterizing the white matter hyperintensity penumbra with cerebral blood flow measures. Neuroimage Clin. 8, 224–229. doi: 10.1016/j.nicl.2015.04.012, PMID: 26106546 PMC4473817

[ref50] Sele S. Liem F. Mérillat S. Jäncke L. (2020). Decline variability of cortical and subcortical regions in aging: a longitudinal study. Front. Hum. Neurosci. 14:14. doi: 10.3389/fnhum.2020.00363, PMID: 33100991 PMC7500514

[ref51] Shabir O. Berwick J. Francis S. E. (2018). Neurovascular dysfunction in vascular dementia, Alzheimer's and atherosclerosis. BMC Neurosci. 19:62. doi: 10.1186/s12868-018-0465-5 30333009 PMC6192291

[ref52] Smirnov M. Destrieux C. Maldonado I. L. (2021). Cerebral white matter vasculature: still uncharted? Brain 144, 3561–3575. doi: 10.1093/brain/awab273, PMID: 34718425

[ref53] Smith S. M. Jenkinson M. Johansen-Berg H. Rueckert D. Nichols T. E. Mackay C. E.. (2006). Tract-based spatial statistics: Voxelwise analysis of multi-subject diffusion data. NeuroImage 31, 1487–1505. doi: 10.1016/j.neuroimage.2006.02.024, PMID: 16624579

[ref54] Smith S. M. Jenkinson M. Woolrich M. W. Beckmann C. F. Behrens T. E. J. Johansen-Berg H.. (2004). Advances in functional and structural MR image analysis and implementation as FSL. NeuroImage 23, S208–S219. doi: 10.1016/j.neuroimage.2004.07.051, PMID: 15501092

[ref55] Soares J. M. Marques P. Alves V. Sousa N. (2013). A hitchhiker's guide to diffusion tensor imaging. Front. Neurosci. 7:31. doi: 10.3389/fnins.2013.00031, PMID: 23486659 PMC3594764

[ref56] Staffaroni A. M. Cobigo Y. Elahi F. M. Casaletto K. B. Walters S. M. Wolf A.. (2019). A longitudinal characterization of perfusion in the aging brain and associations with cognition and neural structure. Hum. Brain Mapp. 40, 3522–3533. doi: 10.1002/hbm.24613, PMID: 31062904 PMC6693488

[ref57] Stefanidis K. B. Askew C. D. Klein T. Lagopoulos J. Summers M. J. (2019). Healthy aging affects cerebrovascular reactivity and pressure-flow responses, but not neurovascular coupling: a cross-sectional study. PLoS One 14:e0217082. doi: 10.1371/journal.pone.0217082, PMID: 31095646 PMC6522028

[ref58] Su F. Y. Peng S. L. (2024). Range and variability of CBF in young adults: PC-MRI and ASL studies. Int. J. Imaging Syst. Technol. 34:e22986. doi: 10.1002/ima.22986

[ref59] Tarumi T. Khan M. A. Liu J. Tseng B. Y. Parker R. Riley J.. (2014). Cerebral hemodynamics in normal aging: central artery stiffness, wave reflection, and pressure pulsatility. J. Cereb. Blood Flow Metab. 34, 971–978. doi: 10.1038/jcbfm.2014.44 24643081 PMC4050241

[ref60] Taylor C. A. Draney M. T. (2004). Experimental and computational methods in cardiovascular fluid mechanics. Annu. Rev. Fluid Mech. 36, 197–231. doi: 10.1146/annurev.fluid.36.050802.121944

[ref61] Thambisetty M. Wan J. Carass A. An Y. Prince J. L. Resnick S. M. (2010). Longitudinal changes in cortical thickness associated with normal aging. NeuroImage 52, 1215–1223. doi: 10.1016/j.neuroimage.2010.04.258, PMID: 20441796 PMC2910226

[ref62] Thore C. R. Anstrom J. A. Moody D. M. Challa V. R. Marion M. C. Brown W. R. (2007). Morphometric analysis of arteriolar tortuosity in human cerebral white matter of preterm, young, and aged subjects. J. Neuropathol. Exp. Neurol. 66, 337–345. doi: 10.1097/nen.0b013e3180537147, PMID: 17483690

[ref63] Turney I. C. Lao P. J. Renteria M. A. Igwe K. C. Berroa J. Rivera A.. (2022). Brain aging among racially and ethnically diverse middle-aged and older adults. JAMA Neurol. 80:73. doi: 10.1001/jamaneurol.2022.3919, PMID: 36374494 PMC9664371

[ref64] Vernooij M. W. van der Lugt A. Ikram M. A. Wielopolski P. A. Vrooman H. A. Hofman A.. (2008). Total cerebral blood flow and total brain perfusion in the general population: the Rotterdam scan study. J. Cereb. Blood Flow Metab. 28, 412–419. doi: 10.1038/sj.jcbfm.9600526, PMID: 17622253

[ref65] Wang J. X. Hill-Jarrett T. Buto P. Pederson A. Sims K. D. Zimmerman S. C.. (2024). Comparison of approaches to control for intracranial volume in research on the association of brain volumes with cognitive outcomes. Hum. Brain Mapp. 45:e26633. doi: 10.1002/hbm.26633 38433682 PMC10910271

[ref66] Wardlaw J. M. Smith C. Dichgans M. (2019). Small vessel disease: mechanisms and clinical implications. Lancet Neurol. 18, 684–696. doi: 10.1016/S1474-4422(19)30079-1, PMID: 31097385

[ref67] Wentland A. L. Grist T. M. Wieben O. (2013). Repeatability and internal consistency of abdominal 2D and 4D phase contrast MR flow measurements. Acad. Radiol. 20, 699–704. doi: 10.1016/j.acra.2012.12.019, PMID: 23510798 PMC3897393

[ref68] Wong E. C. Cronin M. Wu W. C. Inglis B. Frank L. R. Liu T. T. (2006). Velocity-selective arterial spin labeling. Magn. Reson. Med. 55, 1334–1341. doi: 10.1002/mrm.20906, PMID: 16700025

[ref69] Wu C. Honarmand A. R. Schnell S. Kuhn R. Schoeneman S. E. Ansari S. A.. (2016). Age-related changes of Normal cerebral and cardiac blood flow in children and adults aged 7 months to 61 years. J. Am. Heart Assoc. 5:e002657. doi: 10.1161/JAHA.115.002657, PMID: 26727967 PMC4859381

[ref70] Wu W. C. St Lawrence K. S. Licht D. J. Wang D. J. (2010). Quantification issues in arterial spin labeling perfusion magnetic resonance imaging. Top. Magn. Reson. Imaging 21, 65–73. doi: 10.1097/RMR.0b013e31821e570a, PMID: 21613872

[ref71] Zananiri F. V. Jackson P. C. Goddard P. R. Davies E. R. Wells P. N. T. (1991). An evaluation of the accuracy of flow measurements using magnetic-resonance-imaging (Mri). J. Med. Eng. Technol. 15, 170–176. doi: 10.3109/03091909109023704, PMID: 1800748

[ref72] Zarrinkoob L. Ambarki K. Wahlin A. Birgander R. Eklund A. Malm J. (2015). Blood flow distribution in cerebral arteries. J. Cereb. Blood Flow Metab. 35, 648–654. doi: 10.1038/jcbfm.2014.241, PMID: 25564234 PMC4420884

[ref73] Zhao M. Amin-Hanjani S. Ruland S. Curcio A. P. Ostergren L. Charbel F. T. (2007). Regional cerebral blood flow using quantitative MR angiography. Am. J. Neuroradiol. 28, 1470–1473. doi: 10.3174/ajnr.A0582, PMID: 17846193 PMC8134363

[ref74] Zonneveld H. I. Loehrer E. A. Hofman A. Niessen W. J. van der Lugt A. Krestin G. P.. (2015). The bidirectional association between reduced cerebral blood flow and brain atrophy in the general population. J. Cereb. Blood Flow Metab. 35, 1882–1887. doi: 10.1038/jcbfm.2015.157, PMID: 26154865 PMC4635245

