# Supplementary material for: Phase contrast-derived cerebral blood flow is associated with neurodegeneration and cerebrovascular injury in older adults
Source: Front Neurosci. 2025 Jul 4;19:1538956. doi: 10.3389/fnins.2025.1538956 (PMC12271140; doi:10.3389/fnins.2025.1538956)
Supplement: Supplementary file 1 [file Data_Sheet_1.docx]

Table of Contents

[Section 1: Phase Contrast and ASL Agreement: 2](#_Toc199689146)

[Linear Association: 2](#_Toc199689147)

[Bland-Altman Analysis: 3](#_Toc199689148)

[Section 2: Cortical Thickness Associations: 4](#_Toc199689149)

[Forest Plot: 4](#_Toc199689150)

[Total Flow – ASL: 5](#_Toc199689151)

[Total Flow – Phase Contrast: 7](#_Toc199689152)

[Anterior Flow – Phase Contrast: 9](#_Toc199689153)

[Posterior Flow – Phase Contrast: 11](#_Toc199689154)

[Section 3: Regional Volume Associations: 13](#_Toc199689155)

[Forest Plot: 13](#_Toc199689156)

[Total Flow – ASL: 14](#_Toc199689157)

[Total Flow – Phase Contrast: 16](#_Toc199689158)

[Anterior Flow – Phase Contrast: 18](#_Toc199689159)

[Posterior Flow – Phase Contrast: 20](#_Toc199689160)

[Section 4: White Matter Tract Associations: 22](#_Toc199689161)

[Forest Plot: 22](#_Toc199689162)

[Total Flow – ASL: 23](#_Toc199689163)

[Total Flow – Phase Contrast: 24](#_Toc199689164)

[Anterior Flow – Phase Contrast: 25](#_Toc199689165)

[Posterior Flow – Phase Contrast: 26](#_Toc199689166)

[Section 5: White Matter Hyperintensity Associations: 27](#_Toc199689167)

[Scatter Plots: 27](#_Toc199689168)

[Section 6: Age Associations: 28](#_Toc199689169)

[Scatter Plots: 28](#_Toc199689170)

# Section 1: Phase Contrast and ASL Agreement:

## Linear Association:


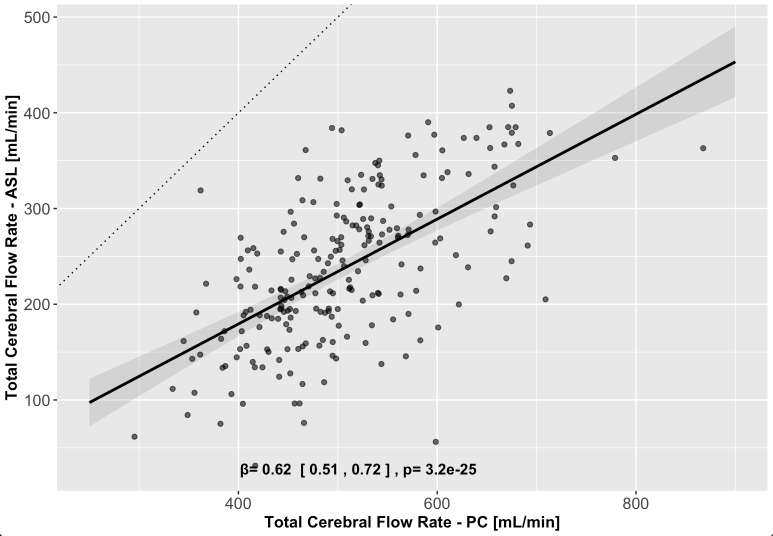


Figure S1: Scatter plot displaying the association between extracranially-derived phase contrast and intracranially-derived arterial spin labeling total cerebral blood flow measurements (n=227). The solid line indicates the linear regression curve, with 95% confidence intervals, between the two measurement methodologies ($\beta$ = 0.62, 95% CI [0.51, 0.72], p < 0.05). The dotted line is an identity line provided for reference.

## Bland-Altman Analysis:


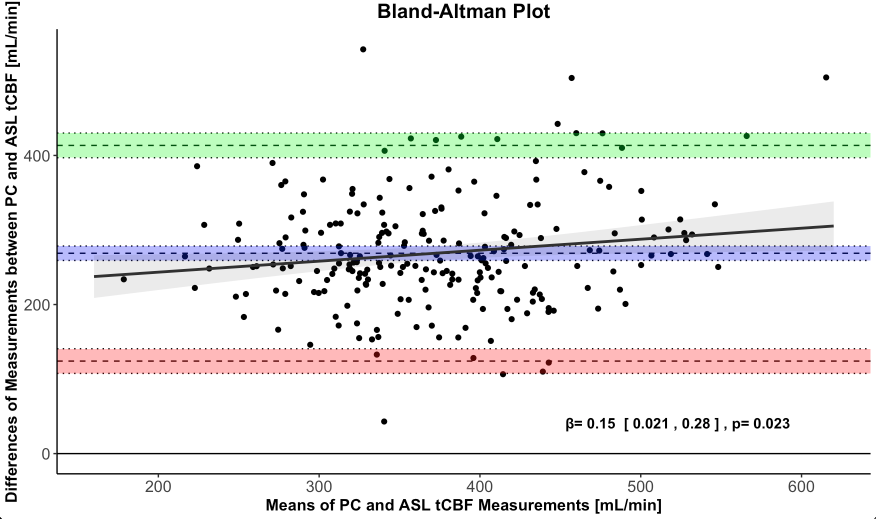


Figure S2: A Bland-Altman analysis testing for proportional bias between phase contrast and pseudo-continuous ASL-derived total cerebral blood flow measures (n=227). The blue shaded region indicates the mean bias with confidence intervals between the measurement methods (268.78 mL/min, 95% CI [259.13, 278.44]). Red and green shaded regions indicate lower and upper, respectively, limits of agreement with confidence intervals between the measurement methods ($LoA_{lower}=$124.13 mL/min, 95% $CI_{lower}$ [107.61, 140.65], $LoA_{upper}=$413.45 mL/min, 95% $CI_{upper}$ [396.93, 429.97]). The black line indicates a significant proportional bias between the measurement methods ($\beta$ = 0.15, 95% CI [0.02, 0.28], p < 0.05).

# Section 2: Cortical Thickness Associations:

## Forest Plot:

Figure S3: Summary forest plot showing the effect sizes of the relationship of intracranial total (n=227), extracranial total (n=232), extracranial anterior (n=292), and extracranial posterior (n=241) cerebral blood flow with cortical thickness. Analyses are adjusted for age, vascular risk factors, intracranial volume, and sex/gender. Each bar represents the results of a separate linear model. Only regions that are perfused by the supplying artery are tested for associations. Filled and open circles represent significant and non-significant associations, respectively.

## Total Flow – ASL:

| Cortical Thickness Region | *b** | *p* | *R*^2^ | 95% CI |
| --- | --- | --- | --- | --- |
| Banks of Superior Temporal Sulcus | 0.09 | .210 | .09 | [-0.05, 0.22] |
| Caudal Anterior Cingulate | 0.10 | .155 | .12 | [-0.04, 0.23] |
| Caudal Middle Frontal | 0.08 | .267 | .06 | [-0.06, 0.21] |
| Cuneus | 0.02 | .779 | .03 | [-0.12, 0.16] |
| **Entorhinal** | **-0.15** | **.015*** | **.23** | **[-0.28, -0.03]** |
| Frontal Pole | 0.04 | .557 | .09 | [-0.09, 0.17] |
| Fusiform | 0.06 | .343 | .15 | [-0.07, 0.19] |
| Inferior Parietal | 0.04 | .576 | .14 | [-0.09, 0.17] |
| Inferior Temporal | -0.06 | .317 | .18 | [-0.19, 0.06] |
| Insula | -0.10 | .159 | .11 | [-0.23, 0.04] |
| **Isthmus Cingulate** | **0.17** | **.013*** | **.09** | **[0.04, 0.31]** |
| Lateral Occipital | 0.00 | .953 | .08 | [-0.13, 0.14] |
| Lateral Orbitofrontal | 0.01 | .864 | .11 | [-0.12, 0.14] |
| Lingual | -0.00 | .966 | .03 | [-0.14, 0.14] |
| Medial Orbitofrontal | 0.12 | .084 | .09 | [-0.02, 0.25] |
| Middle Temporal | -0.03 | .644 | .16 | [-0.16, 0.10] |
| Paracentral | -0.08 | .266 | .04 | [-0.22, 0.06] |
| Parahippocampal | 0.01 | .913 | .04 | [-0.13, 0.14] |
| Pars Opercularis | 0.01 | .868 | .04 | [-0.13, 0.15] |
| Pars Orbitalis | 0.01 | .917 | .14 | [-0.12, 0.14] |
| Pars Triangularis | 0.07 | .338 | .10 | [-0.07, 0.20] |
| Pericalcarine | 0.11 | .114 | .02 | [-0.03, 0.25] |
| Postcentral | -0.03 | .613 | .14 | [-0.16, 0.10] |
| Posterior Cingulate | 0.01 | .837 | .06 | [-0.12, 0.15] |
| Precentral | 0.07 | .255 | .17 | [-0.05, 0.20] |
| Precuneus | -0.06 | .411 | .08 | [-0.19, 0.08] |
| Rostral Anterior Cingulate | -0.02 | .787 | .06 | [-0.15, 0.12] |
| Rostral Middle Frontal | 0.04 | .526 | .07 | [-0.09, 0.18] |
| Superior Frontal | 0.10 | .145 | .11 | [-0.03, 0.23] |
| Superior Parietal | -0.08 | .213 | .10 | [-0.22, 0.05] |
| Superior Temporal | -0.03 | .693 | .20 | [-0.15, 0.10] |
| Supramarginal | 0.04 | .548 | .19 | [-0.09, 0.17] |
| Temporal Pole | -0.13 | .053 | .17 | [-0.25, 0.00] |
| Transverse Temporal | 0.10 | .119 | .16 | [-0.03, 0.23] |
| *Note.* The data was corrected for multiple comparisons. b* and 95% CI refer to standardized effect sizes. Analyses are adjusted for age, vascular risk factors, intracranial volume, and sex. | | | | |
| * p < .05, ** p < .01, *** p < .001 | | | | |

## Total Flow – Phase Contrast:

| Cortical Thickness Region | *b** | *p* | *R*^2^ | 95% CI |
| --- | --- | --- | --- | --- |
| Banks of Superior Temporal Sulcus | 0.02 | .737 | .09 | [-0.11, 0.16] |
| Caudal Anterior Cingulate | 0.07 | .284 | .12 | [-0.06, 0.21] |
| Caudal Middle Frontal | 0.04 | .553 | .06 | [-0.10, 0.18] |
| Cuneus | -0.09 | .206 | .04 | [-0.23, 0.05] |
| Entorhinal | -0.12 | .071 | .21 | [-0.24, 0.01] |
| Frontal Pole | 0.03 | .686 | .08 | [-0.11, 0.16] |
| Fusiform | -0.00 | .991 | .15 | [-0.13, 0.13] |
| Inferior Parietal | 0.02 | .794 | .13 | [-0.11, 0.15] |
| Inferior Temporal | -0.01 | .899 | .16 | [-0.14, 0.12] |
| Insula | -0.12 | .074 | .11 | [-0.26, 0.01] |
| Isthmus Cingulate | 0.07 | .339 | .07 | [-0.07, 0.20] |
| Lateral Occipital | -0.03 | .647 | .08 | [-0.17, 0.10] |
| Lateral Orbitofrontal | 0.00 | .960 | .11 | [-0.13, 0.14] |
| Lingual | -0.11 | .136 | .04 | [-0.25, 0.03] |
| Medial Orbitofrontal | 0.03 | .704 | .08 | [-0.11, 0.16] |
| Middle Temporal | -0.09 | .193 | .15 | [-0.22, 0.04] |
| Paracentral | -0.11 | .120 | .04 | [-0.25, 0.03] |
| Parahippocampal | -0.00 | .946 | .04 | [-0.14, 0.13] |
| Pars Opercularis | -0.03 | .632 | .04 | [-0.17, 0.11] |
| Pars Orbitalis | -0.12 | .083 | .15 | [-0.25, 0.01] |
| Pars Triangularis | -0.11 | .109 | .10 | [-0.25, 0.02] |
| Pericalcarine | 0.03 | .706 | .01 | [-0.11, 0.17] |
| Postcentral | -0.08 | .242 | .14 | [-0.21, 0.05] |
| Posterior Cingulate | 0.06 | .421 | .06 | [-0.08, 0.19] |
| Precentral | 0.04 | .540 | .15 | [-0.09, 0.17] |
| Precuneus | -0.02 | .787 | .07 | [-0.16, 0.12] |
| Rostral Anterior Cingulate | -0.01 | .890 | .06 | [-0.15, 0.13] |
| Rostral Middle Frontal | -0.02 | .801 | .06 | [-0.15, 0.12] |
| Superior Frontal | 0.06 | .419 | .09 | [-0.08, 0.19] |
| Superior Parietal | -0.05 | .427 | .09 | [-0.19, 0.08] |
| Superior Temporal | -0.07 | .280 | .19 | [-0.20, 0.06] |
| Supramarginal | -0.03 | .596 | .17 | [-0.16, 0.09] |
| Temporal Pole | -0.06 | .408 | .15 | [-0.19, 0.08] |
| Transverse Temporal | -0.07 | .295 | .15 | [-0.20, 0.06] |
| *Note.* The data was corrected for multiple comparisons. b* and 95% CI refer to standardized effect sizes. Analyses are adjusted for age, vascular risk factors, intracranial volume, and sex. | | | | |
| * p < .05, ** p < .01, *** p < .001 | | | | |

## Anterior Flow – Phase Contrast:

| Cortical Thickness Region | *b** | *p* | *R*^2^ | 95% CI |
| --- | --- | --- | --- | --- |
| Banks of Superior Temporal Sulcus | 0.01 | .831 | .09 | [-0.10, 0.13] |
| Caudal Anterior Cingulate | -0.01 | .883 | .14 | [-0.12, 0.11] |
| Caudal Middle Frontal | 0.05 | .429 | .07 | [-0.07, 0.17] |
| Cuneus |  |  |  |  |
| Entorhinal | -0.09 | .132 | .20 | [-0.20, 0.03] |
| Frontal Pole | 0.02 | .706 | .07 | [-0.10, 0.14] |
| Fusiform |  |  |  |  |
| Inferior Parietal | 0.05 | .356 | .15 | [-0.06, 0.17] |
| Inferior Temporal | -0.00 | .979 | .16 | [-0.11, 0.11] |
| Insula | -0.10 | .109 | .07 | [-0.22, 0.02] |
| Isthmus Cingulate |  |  |  |  |
| Lateral Occipital | 0.02 | .767 | .08 | [-0.10, 0.14] |
| Lateral Orbitofrontal | 0.02 | .750 | .10 | [-0.10, 0.14] |
| Lingual |  |  |  |  |
| Medial Orbitofrontal | 0.02 | .803 | .07 | [-0.10, 0.13] |
| Middle Temporal | -0.02 | .690 | .16 | [-0.14, 0.09] |
| Paracentral | -0.02 | .698 | .04 | [-0.15, 0.10] |
| Parahippocampal |  |  |  |  |
| Pars Opercularis | 0.01 | .833 | .04 | [-0.11, 0.13] |
| Pars Orbitalis | -0.05 | .398 | .13 | [-0.16, 0.07] |
| Pars Triangularis | -0.06 | .350 | .10 | [-0.17, 0.06] |
| Pericalcarine |  |  |  |  |
| Postcentral | 0.01 | .811 | .14 | [-0.10, 0.13] |
| Posterior Cingulate | 0.06 | .300 | .06 | [-0.06, 0.18] |
| Precentral | 0.10 | .068 | .18 | [-0.01, 0.22] |
| Precuneus | 0.05 | .446 | .08 | [-0.07, 0.16] |
| Rostral Anterior Cingulate | -0.02 | .797 | .08 | [-0.13, 0.10] |
| Rostral Middle Frontal | 0.01 | .814 | .05 | [-0.11, 0.13] |
| Superior Frontal | 0.11 | .074 | .11 | [-0.01, 0.22] |
| Superior Parietal | 0.02 | .757 | .09 | [-0.10, 0.14] |
| Superior Temporal | 0.02 | .699 | .18 | [-0.09, 0.13] |
| Supramarginal | 0.03 | .590 | .17 | [-0.08, 0.14] |
| Temporal Pole | -0.06 | .338 | .14 | [-0.17, 0.06] |
| Transverse Temporal | 0.02 | .671 | .15 | [-0.09, 0.14] |
| *Note.* The data was corrected for multiple comparisons. b* and 95% CI refer to standardized effect sizes. Analyses are adjusted for age, vascular risk factors, intracranial volume, and sex. | | | | |
| * p < .05, ** p < .01, *** p < .001 | | | | |

## Posterior Flow – Phase Contrast:

| Cortical Thickness Region | *b** | *p* | *R*^2^ | 95% CI |
| --- | --- | --- | --- | --- |
| Banks of Superior Temporal Sulcus |  |  |  |  |
| Caudal Anterior Cingulate |  |  |  |  |
| Caudal Middle Frontal |  |  |  |  |
| Cuneus | -0.03 | .699 | .03 | [-0.16, 0.11] |
| Entorhinal |  |  |  |  |
| Frontal Pole |  |  |  |  |
| Fusiform | -0.03 | .623 | .14 | [-0.15, 0.09] |
| Inferior Parietal |  |  |  |  |
| Inferior Temporal | 0.01 | .817 | .14 | [-0.11, 0.14] |
| Insula |  |  |  |  |
| Isthmus Cingulate | 0.07 | .262 | .09 | [-0.05, 0.20] |
| Lateral Occipital | -0.04 | .534 | .07 | [-0.17, 0.09] |
| Lateral Orbitofrontal |  |  |  |  |
| Lingual | -0.06 | .344 | .03 | [-0.19, 0.07] |
| Medial Orbitofrontal |  |  |  |  |
| Middle Temporal |  |  |  |  |
| Paracentral |  |  |  |  |
| Parahippocampal | 0.12 | .062 | .06 | [-0.01, 0.25] |
| Pars Opercularis |  |  |  |  |
| Pars Orbitalis |  |  |  |  |
| Pars Triangularis |  |  |  |  |
| Pericalcarine | 0.02 | .713 | .01 | [-0.11, 0.16] |
| Postcentral |  |  |  |  |
| Posterior Cingulate |  |  |  |  |
| Precentral |  |  |  |  |
| Precuneus | -0.03 | .614 | .07 | [-0.16, 0.10] |
| Rostral Anterior Cingulate |  |  |  |  |
| Rostral Middle Frontal |  |  |  |  |
| Superior Frontal |  |  |  |  |
| Superior Parietal | -0.07 | .306 | .07 | [-0.20, 0.06] |
| Superior Temporal |  |  |  |  |
| Supramarginal |  |  |  |  |
| Temporal Pole |  |  |  |  |
| Transverse Temporal |  |  |  |  |
| *Note.* The data was corrected for multiple comparisons. b* and 95% CI refer to standardized effect sizes. Analyses are adjusted for age, vascular risk factors, intracranial volume, and sex. | | | | |
| * p < .05, ** p < .01, *** p < .001 | | | | |

# Section 3: Regional Volume Associations:

## Forest Plot:

Figure S4: Summary forest plot showing the effect sizes of the relationship of intracranial total (n=227), extracranial total (n=232), extracranial anterior (n=292), and extracranial posterior (n=241) cerebral blood flow with regional volumes. Analyses are adjusted for age, vascular risk factors, intracranial volume, and sex/gender. Each bar represents the results of a separate linear model. Organized by cortical, subcortical, and ventricular system regions. Only regions that are perfused by the supplying artery are tested for associations. Filled and open circles represent significant and non-significant associations, respectively.

## Total Flow – ASL:

| Regional Thickness | *b** | *p* | *R*^2^ | 95% CI |
| --- | --- | --- | --- | --- |
| 3rd Ventricle | -0.09 | .107 | .35 | [-0.21, 0.02] |
| 4th Ventricle | -0.03 | .631 | .14 | [-0.16, 0.10] |
| Accumbens Area | 0.04 | .511 | .20 | [-0.08, 0.17] |
| Amygdala | -0.02 | .782 | .32 | [-0.13, 0.10] |
| Banks of Superior Temporal Sulcus | 0.07 | .281 | .24 | [-0.05, 0.19] |
| **Caudal Anterior Cingulate** | **0.16** | **.012*** | **.21** | **[0.04, 0.29]** |
| Caudal Middle Frontal | -0.05 | .452 | .29 | [-0.16, 0.07] |
| Caudate | -0.03 | .651 | .11 | [-0.16, 0.10] |
| **Cerebellum Cortex** | **0.15** | **.008**** | **.38** | **[0.04, 0.26]** |
| Cuneus | 0.06 | .311 | .29 | [-0.06, 0.18] |
| **Entorhinal** | **-0.13** | **.032*** | **.27** | **[-0.25, -0.01]** |
| Frontal Pole | 0.02 | .762 | .08 | [-0.11, 0.16] |
| Fusiform | 0.03 | .515 | .50 | [-0.07, 0.13] |
| Hippocampus | -0.01 | .843 | .28 | [-0.13, 0.11] |
| Inferior Lateral Ventricle | -0.05 | .360 | .34 | [-0.17, 0.06] |
| Inferior Parietal | 0.05 | .421 | .32 | [-0.07, 0.16] |
| Inferior Temporal | 0.03 | .539 | .42 | [-0.07, 0.14] |
| Insula | -0.05 | .332 | .44 | [-0.16, 0.05] |
| Isthmus Cingulate | -0.01 | .865 | .34 | [-0.12, 0.10] |
| Lateral Occipital | 0.01 | .857 | .38 | [-0.10, 0.12] |
| Lateral Orbitofrontal | 0.01 | .786 | .50 | [-0.09, 0.11] |
| Lateral Ventricle | -0.10 | .078 | .33 | [-0.22, 0.01] |
| Lingual | 0.00 | .956 | .26 | [-0.12, 0.12] |
| Medial Orbitofrontal | -0.04 | .422 | .43 | [-0.15, 0.06] |
| Middle Temporal | 0.03 | .578 | .40 | [-0.08, 0.14] |
| Pallidum | -0.03 | .672 | .23 | [-0.15, 0.10] |
| Paracentral | 0.02 | .722 | .38 | [-0.09, 0.13] |
| Parahippocampal | -0.01 | .851 | .21 | [-0.14, 0.11] |
| Pars Opercularis | -0.02 | .708 | .23 | [-0.15, 0.10] |
| Pars Orbitalis | -0.01 | .891 | .27 | [-0.13, 0.11] |
| Pars Triangularis | -0.01 | .892 | .19 | [-0.14, 0.12] |
| Pericalcarine | 0.11 | .099 | .15 | [-0.02, 0.24] |
| Postcentral | 0.03 | .530 | .41 | [-0.07, 0.14] |
| Posterior Cingulate | 0.06 | .330 | .27 | [-0.06, 0.18] |
| Precentral | 0.07 | .169 | .47 | [-0.03, 0.17] |
| Precuneus | 0.03 | .550 | .43 | [-0.07, 0.14] |
| Putamen | 0.04 | .514 | .09 | [-0.09, 0.18] |
| Rostral Anterior Cingulate | -0.07 | .212 | .30 | [-0.19, 0.04] |
| Rostral Middle Frontal | 0.04 | .511 | .36 | [-0.07, 0.15] |
| Superior Frontal | -0.02 | .726 | .49 | [-0.12, 0.08] |
| Superior Parietal | 0.05 | .420 | .38 | [-0.07, 0.16] |
| Superior Temporal | -0.04 | .467 | .40 | [-0.15, 0.07] |
| Supramarginal | 0.03 | .536 | .38 | [-0.08, 0.15] |
| Temporal Pole | -0.06 | .351 | .20 | [-0.19, 0.07] |
| Transverse Temporal | 0.06 | .308 | .30 | [-0.06, 0.18] |
| Ventral Diencephalon | 0.07 | .237 | .35 | [-0.04, 0.18] |
| *Note.* The data was corrected for multiple comparisons. b* and 95% CI refer to standardized effect sizes. Analyses are adjusted for age, vascular risk factors, intracranial volume, and sex. | | | | |
| * p < .05, ** p < .01, *** p < .001 | | | | |

## Total Flow – Phase Contrast:

| Regional Thickness | *b** | *p* | *R*^2^ | 95% CI |
| --- | --- | --- | --- | --- |
| **3rd Ventricle** | **-0.20** | **< .001***** | **.38** | **[-0.32, -0.09]** |
| **4th Ventricle** | **-0.19** | **.005**** | **.16** | **[-0.32, -0.06]** |
| Accumbens Area | 0.12 | .059 | .22 | [-0.00, 0.25] |
| Amygdala | 0.05 | .424 | .33 | [-0.07, 0.16] |
| Banks of Superior Temporal Sulcus | 0.08 | .223 | .26 | [-0.05, 0.20] |
| **Caudal Anterior Cingulate** | **0.19** | **.004**** | **.20** | **[0.06, 0.31]** |
| Caudal Middle Frontal | -0.01 | .922 | .30 | [-0.12, 0.11] |
| Caudate | 0.06 | .414 | .12 | [-0.08, 0.19] |
| Cerebellum Cortex | 0.08 | .172 | .37 | [-0.03, 0.19] |
| Cuneus | 0.10 | .115 | .30 | [-0.02, 0.21] |
| Entorhinal | 0.03 | .639 | .26 | [-0.09, 0.15] |
| Frontal Pole | 0.04 | .554 | .09 | [-0.09, 0.18] |
| Fusiform | 0.08 | .140 | .51 | [-0.02, 0.17] |
| Hippocampus | 0.06 | .357 | .29 | [-0.06, 0.18] |
| Inferior Lateral Ventricle | -0.10 | .089 | .34 | [-0.22, 0.01] |
| Inferior Parietal | 0.10 | .097 | .33 | [-0.02, 0.21] |
| Inferior Temporal | 0.04 | .415 | .42 | [-0.06, 0.15] |
| Insula | 0.07 | .165 | .46 | [-0.03, 0.18] |
| Isthmus Cingulate | 0.03 | .626 | .35 | [-0.09, 0.14] |
| Lateral Occipital | 0.03 | .542 | .39 | [-0.08, 0.14] |
| **Lateral Orbitofrontal** | **0.11** | **.024*** | **.54** | **[0.02, 0.21]** |
| **Lateral Ventricle** | **-0.13** | **.027*** | **.34** | **[-0.25, -0.02]** |
| Lingual | 0.02 | .784 | .26 | [-0.10, 0.14] |
| Medial Orbitofrontal | -0.01 | .869 | .45 | [-0.11, 0.10] |
| Middle Temporal | -0.02 | .751 | .41 | [-0.13, 0.09] |
| Pallidum | -0.04 | .537 | .24 | [-0.16, 0.08] |
| Paracentral | 0.02 | .740 | .37 | [-0.09, 0.13] |
| Parahippocampal | 0.05 | .476 | .23 | [-0.08, 0.17] |
| Pars Opercularis | 0.09 | .132 | .26 | [-0.03, 0.22] |
| Pars Orbitalis | 0.08 | .178 | .30 | [-0.04, 0.20] |
| Pars Triangularis | -0.00 | .952 | .21 | [-0.13, 0.12] |
| **Pericalcarine** | **0.14** | **.034*** | **.16** | **[0.01, 0.27]** |
| Postcentral | 0.03 | .564 | .41 | [-0.08, 0.14] |
| Posterior Cingulate | 0.12 | .047* | .28 | [0.00, 0.24] |
| Precentral | 0.09 | .074 | .49 | [-0.01, 0.19] |
| **Precuneus** | **0.17** | **.001**** | **.47** | **[0.07, 0.28]** |
| Putamen | 0.06 | .422 | .10 | [-0.08, 0.19] |
| Rostral Anterior Cingulate | 0.05 | .451 | .30 | [-0.07, 0.16] |
| Rostral Middle Frontal | 0.07 | .195 | .38 | [-0.04, 0.19] |
| Superior Frontal | 0.06 | .277 | .51 | [-0.04, 0.15] |
| **Superior Parietal** | **0.13** | **.020*** | **.40** | **[0.02, 0.24]** |
| Superior Temporal | 0.07 | .218 | .42 | [-0.04, 0.18] |
| Supramarginal | 0.05 | .351 | .40 | [-0.06, 0.16] |
| Temporal Pole | 0.07 | .275 | .20 | [-0.06, 0.20] |
| Transverse Temporal | 0.04 | .510 | .32 | [-0.08, 0.16] |
| Ventral Diencephalon | 0.10 | .098 | .36 | [-0.02, 0.21] |
| *Note.* The data was corrected for multiple comparisons. b* and 95% CI refer to standardized effect sizes. Analyses are adjusted for age, vascular risk factors, intracranial volume, and sex. | | | | |
| * p < .05, ** p < .01, *** p < .001 | | | | |

## Anterior Flow – Phase Contrast:

| Regional Thickness | *b** | *p* | *R*^2^ | 95% CI |
| --- | --- | --- | --- | --- |
| 3rd Ventricle |  |  |  |  |
| 4th Ventricle |  |  |  |  |
| **Accumbens Area** | **0.11** | **.041*** | **.24** | **[0.01, 0.22]** |
| Amygdala | 0.06 | .249 | .33 | [-0.04, 0.16] |
| Banks of Superior Temporal Sulcus | 0.09 | .099 | .28 | [-0.02, 0.19] |
| **Caudal Anterior Cingulate** | **0.13** | **.021*** | **.17** | **[0.02, 0.25]** |
| Caudal Middle Frontal | 0.01 | .852 | .34 | [-0.09, 0.11] |
| Caudate | 0.07 | .232 | .15 | [-0.04, 0.18] |
| Cerebellum Cortex |  |  |  |  |
| Cuneus |  |  |  |  |
| Entorhinal | 0.04 | .478 | .24 | [-0.07, 0.15] |
| Frontal Pole | 0.03 | .582 | .08 | [-0.09, 0.15] |
| Fusiform |  |  |  |  |
| Hippocampus |  |  |  |  |
| Inferior Lateral Ventricle |  |  |  |  |
| **Inferior Parietal** | **0.12** | **.017*** | **.36** | **[0.02, 0.22]** |
| Inferior Temporal | 0.08 | .101 | .44 | [-0.01, 0.17] |
| Insula | 0.06 | .198 | .46 | [-0.03, 0.15] |
| Isthmus Cingulate |  |  |  |  |
| Lateral Occipital | 0.06 | .248 | .39 | [-0.04, 0.15] |
| **Lateral Orbitofrontal** | **0.10** | **.020*** | **.54** | **[0.02, 0.18]** |
| Lateral Ventricle |  |  |  |  |
| Lingual |  |  |  |  |
| Medial Orbitofrontal | 0.00 | .993 | .45 | [-0.09, 0.09] |
| Middle Temporal | 0.04 | .412 | .44 | [-0.05, 0.13] |
| Pallidum | 0.04 | .500 | .24 | [-0.07, 0.15] |
| Paracentral | 0.08 | .110 | .39 | [-0.02, 0.18] |
| Parahippocampal |  |  |  |  |
| Pars Opercularis | 0.08 | .129 | .28 | [-0.02, 0.19] |
| Pars Orbitalis | 0.10 | .057 | .31 | [-0.00, 0.20] |
| Pars Triangularis | 0.07 | .240 | .21 | [-0.04, 0.18] |
| Pericalcarine |  |  |  |  |
| **Postcentral** | **0.10** | **.035*** | **.42** | **[0.01, 0.20]** |
| **Posterior Cingulate** | **0.13** | **.012*** | **.29** | **[0.03, 0.24]** |
| **Precentral** | **0.14** | **.002**** | **.52** | **[0.05, 0.23]** |
| **Precuneus** | **0.15** | **.002**** | **.47** | **[0.06, 0.24]** |
| Putamen | 0.11 | .069 | .14 | [-0.01, 0.22] |
| Rostral Anterior Cingulate | 0.03 | .538 | .31 | [-0.07, 0.14] |
| **Rostral Middle Frontal** | **0.13** | **.009**** | **.40** | **[0.03, 0.22]** |
| **Superior Frontal** | **0.10** | **.029*** | **.52** | **[0.01, 0.18]** |
| Superior Parietal | 0.10 | .052 | .40 | [-0.00, 0.19] |
| Superior Temporal | 0.10 | .052 | .40 | [-0.00, 0.19] |
| Supramarginal | 0.05 | .330 | .39 | [-0.05, 0.14] |
| Temporal Pole | 0.09 | .130 | .20 | [-0.02, 0.20] |
| Transverse Temporal | 0.06 | .258 | .32 | [-0.04, 0.16] |
| Ventral Diencephalon |  |  |  |  |
| *Note.* The data was corrected for multiple comparisons. b* and 95% CI refer to standardized effect sizes. Analyses are adjusted for age, vascular risk factors, intracranial volume, and sex. | | | | |
| * p < .05, ** p < .01, *** p < .001 | | | | |

## Posterior Flow – Phase Contrast:

| Regional Thickness | *b** | *p* | *R*^2^ | 95% CI |
| --- | --- | --- | --- | --- |
| 3rd Ventricle |  |  |  |  |
| 4th Ventricle |  |  |  |  |
| Accumbens Area |  |  |  |  |
| Amygdala |  |  |  |  |
| Banks of Superior Temporal Sulcus |  |  |  |  |
| Caudal Anterior Cingulate |  |  |  |  |
| Caudal Middle Frontal |  |  |  |  |
| Caudate |  |  |  |  |
| **Cerebellum Cortex** | **0.13** | **.017*** | **.37** | **[0.02, 0.24]** |
| **Cuneus** | **0.13** | **.024*** | **.30** | **[0.02, 0.24]** |
| Entorhinal |  |  |  |  |
| Frontal Pole |  |  |  |  |
| Fusiform | 0.04 | .444 | .52 | [-0.06, 0.13] |
| Hippocampus | 0.09 | .098 | .30 | [-0.02, 0.21] |
| Inferior Lateral Ventricle |  |  |  |  |
| Inferior Parietal |  |  |  |  |
| Inferior Temporal | -0.01 | .882 | .41 | [-0.11, 0.10] |
| Insula |  |  |  |  |
| Isthmus Cingulate | 0.09 | .099 | .36 | [-0.02, 0.20] |
| Lateral Occipital | 0.01 | .796 | .38 | [-0.09, 0.12] |
| Lateral Orbitofrontal |  |  |  |  |
| Lateral Ventricle |  |  |  |  |
| Lingual | 0.03 | .623 | .24 | [-0.09, 0.15] |
| Medial Orbitofrontal |  |  |  |  |
| Middle Temporal |  |  |  |  |
| Pallidum |  |  |  |  |
| Paracentral |  |  |  |  |
| **Parahippocampal** | **0.15** | **.014*** | **.25** | **[0.03, 0.26]** |
| Pars Opercularis |  |  |  |  |
| Pars Orbitalis |  |  |  |  |
| Pars Triangularis |  |  |  |  |
| Pericalcarine | 0.13 | .048* | .14 | [0.00, 0.25] |
| Postcentral |  |  |  |  |
| Posterior Cingulate |  |  |  |  |
| Precentral |  |  |  |  |
| Precuneus | 0.09 | .057 | .47 | [-0.00, 0.19] |
| Putamen |  |  |  |  |
| Rostral Anterior Cingulate |  |  |  |  |
| Rostral Middle Frontal |  |  |  |  |
| Superior Frontal |  |  |  |  |
| Superior Parietal | 0.10 | .051 | .39 | [-0.00, 0.21] |
| Superior Temporal |  |  |  |  |
| Supramarginal |  |  |  |  |
| Temporal Pole |  |  |  |  |
| Transverse Temporal |  |  |  |  |
| Ventral Diencephalon | 0.11 | .050* | .36 | [0.00, 0.21] |
| *Note.* The data was corrected for multiple comparisons. b* and 95% CI refer to standardized effect sizes. Analyses are adjusted for age, vascular risk factors, intracranial volume, and sex. | | | | |
| * p < .05, ** p < .01, *** p < .001 | | | | |

# Section 4: White Matter Tract Associations:

## Forest Plot:

Figure S5: Summary forest plot showing the effect sizes of the relationship of intracranial total (n=227), extracranial total (n=232), extracranial anterior (n=292), and extracranial posterior (n=241) cerebral blood flow with white matter tract integrity. Analyses are adjusted for age, vascular risk factors, intracranial volume, and sex/gender. Each bar represents the results of a separate linear model. Organized by commissural, projection, limbic, and association tracts. Filled and open circles represent significant and non-significant associations, respectively.

## Total Flow – ASL:

| DTI Tracts | *b** | *p* | *R*^2^ | 95% CI |
| --- | --- | --- | --- | --- |
| Anterior Thalamic Radiation | 0.09 | .190 | .11 | [-0.04, 0.22] |
| Cingulum Cingulate Gyrus | 0.06 | .358 | .08 | [-0.07, 0.20] |
| Cingulum Hippocampus | 0.03 | .666 | .09 | [-0.10, 0.16] |
| Corticospinal Tract | -0.09 | .202 | .03 | [-0.23, 0.05] |
| Forceps Major | 0.06 | .315 | .23 | [-0.06, 0.19] |
| Forceps Minor | 0.03 | .690 | .22 | [-0.10, 0.15] |
| Inferior Fronto-occipital Fasciculus | 0.08 | .199 | .17 | [-0.04, 0.21] |
| Inferior Longitudinal Fasciculus | 0.05 | .438 | .11 | [-0.08, 0.18] |
| Superior Longitudinal Fasciculus | 0.10 | .149 | .05 | [-0.04, 0.24] |
| Superior Longitudinal Fasciculus Temporal Part | 0.03 | .686 | .03 | [-0.11, 0.17] |
| Uncinate Fasciculus | 0.06 | .385 | .04 | [-0.08, 0.20] |
| *Note.* The data was corrected for multiple comparisons. b* and 95% CI refer to standardized effect sizes. Analyses are adjusted for age, vascular risk factors, intracranial volume, and sex. | | | | |
| * p < .05, ** p < .01, *** p < .001 | | | | |

## Total Flow – Phase Contrast:

| DTI Tracts | *b** | *p* | *R*^2^ | 95% CI |
| --- | --- | --- | --- | --- |
| **Anterior Thalamic Radiation** | **0.15** | **.027*** | **.13** | **[0.02, 0.28]** |
| **Cingulum Cingulate Gyrus** | **0.18** | **.008**** | **.11** | **[0.05, 0.32]** |
| Cingulum Hippocampus | 0.10 | .130 | .10 | [-0.03, 0.24] |
| Corticospinal Tract | -0.02 | .753 | .02 | [-0.16, 0.12] |
| Forceps Major | 0.09 | .149 | .22 | [-0.03, 0.22] |
| **Forceps Minor** | **0.14** | **.031*** | **.23** | **[0.01, 0.26]** |
| **Inferior Fronto-occipital Fasciculus** | **0.14** | **.033*** | **.17** | **[0.01, 0.27]** |
| Inferior Longitudinal Fasciculus | 0.13 | .052 | .12 | [-0.00, 0.27] |
| Superior Longitudinal Fasciculus | 0.11 | .121 | .05 | [-0.03, 0.25] |
| Superior Longitudinal Fasciculus Temporal Part | 0.05 | .483 | .03 | [-0.09, 0.19] |
| Uncinate Fasciculus | 0.09 | .192 | .05 | [-0.05, 0.23] |
| *Note.* The data was corrected for multiple comparisons. b* and 95% CI refer to standardized effect sizes. Analyses are adjusted for age, vascular risk factors, intracranial volume, and sex. | | | | |
| * p < .05, ** p < .01, *** p < .001 | | | | |

## Anterior Flow – Phase Contrast:

| DTI Tracts | *b** | *p* | *R*^2^ | 95% CI |
| --- | --- | --- | --- | --- |
| **Anterior Thalamic Radiation** | **0.14** | **.024*** | **.11** | **[0.02, 0.25]** |
| **Cingulum Cingulate Gyrus** | **0.17** | **.005**** | **.09** | **[0.05, 0.29]** |
| Cingulum Hippocampus | 0.06 | .335 | .11 | [-0.06, 0.17] |
| Corticospinal Tract | 0.05 | .409 | .02 | [-0.07, 0.17] |
| Forceps Major | 0.11 | .060 | .21 | [-0.00, 0.22] |
| **Forceps Minor** | **0.15** | **.008**** | **.20** | **[0.04, 0.26]** |
| **Inferior Fronto-occipital Fasciculus** | **0.12** | **.036*** | **.15** | **[0.01, 0.24]** |
| **Inferior Longitudinal Fasciculus** | **0.13** | **.033*** | **.11** | **[0.01, 0.24]** |
| Superior Longitudinal Fasciculus | 0.13 | .038* | .04 | [0.01, 0.25] |
| Superior Longitudinal Fasciculus Temporal Part | 0.05 | .388 | .02 | [-0.07, 0.18] |
| Uncinate Fasciculus | 0.12 | .052 | .03 | [-0.00, 0.24] |
| *Note.* The data was corrected for multiple comparisons. b* and 95% CI refer to standardized effect sizes. Analyses are adjusted for age, vascular risk factors, intracranial volume, and sex. | | | | |
| * p < .05, ** p < .01, *** p < .001 | | | | |

## Posterior Flow – Phase Contrast:

| DTI Tracts | *b** | *p* | *R*^2^ | 95% CI |
| --- | --- | --- | --- | --- |
| Anterior Thalamic Radiation | 0.12 | .069 | .13 | [-0.01, 0.24] |
| **Cingulum Cingulate Gyrus** | **0.16** | **.015*** | **.09** | **[0.03, 0.29]** |
| **Cingulum Hippocampus** | **0.21** | **.001**** | **.12** | **[0.08, 0.34]** |
| Corticospinal Tract | -0.01 | .834 | .03 | [-0.15, 0.12] |
| Forceps Major | 0.12 | .059 | .21 | [-0.00, 0.23] |
| **Forceps Minor** | **0.13** | **.037*** | **.21** | **[0.01, 0.25]** |
| **Inferior Fronto-occipital Fasciculus** | **0.15** | **.014*** | **.16** | **[0.03, 0.28]** |
| Inferior Longitudinal Fasciculus | 0.13 | .042* | .11 | [0.01, 0.26] |
| Superior Longitudinal Fasciculus | 0.09 | .200 | .05 | [-0.04, 0.22] |
| Superior Longitudinal Fasciculus Temporal Part | 0.01 | .902 | .03 | [-0.12, 0.14] |
| Uncinate Fasciculus | 0.10 | .130 | .06 | [-0.03, 0.23] |
| *Note.* The data was corrected for multiple comparisons. b* and 95% CI refer to standardized effect sizes. Analyses are adjusted for age, vascular risk factors, intracranial volume, and sex. | | | | |
| * p < .05, ** p < .01, *** p < .001 | | | | |

# Section 5: White Matter Hyperintensity Associations:

## Scatter Plots:


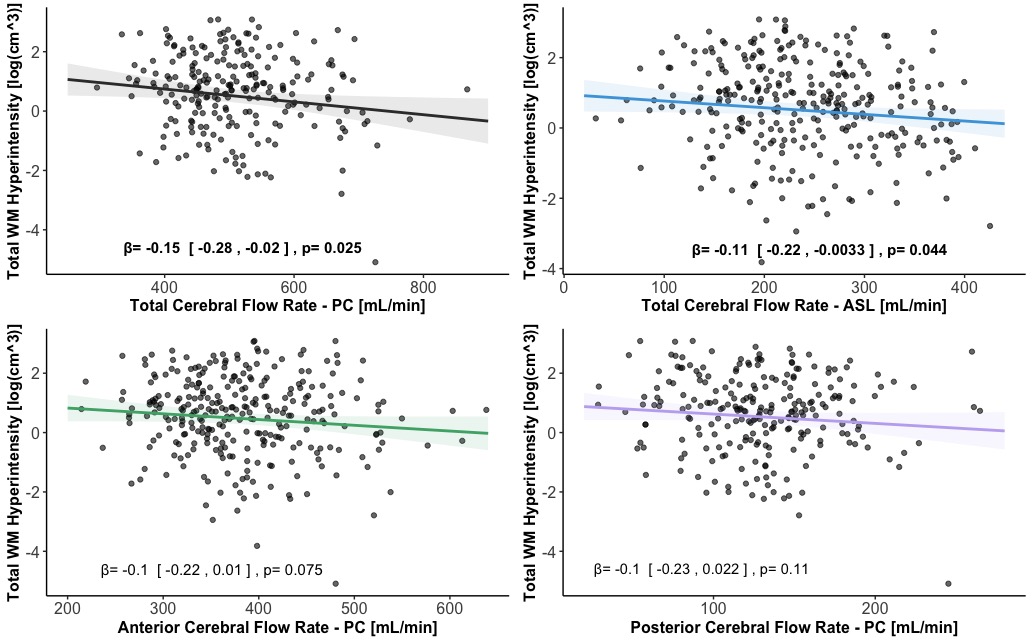


Figure S6: Scatter plots displaying the association between extracranial total (n=232), intracranial total (n=227), extracranial anterior (n=292), and extracranial posterior (n=241) cerebral blood flow and total WMH volume. Linear regression curves, with 95% confidence intervals, are adjusted for age, vascular risk factors, intracranial volume, and sex. Scatter plot datapoints are visualized on unadjusted values.

# Section 6: Age Associations:

## Scatter Plots:


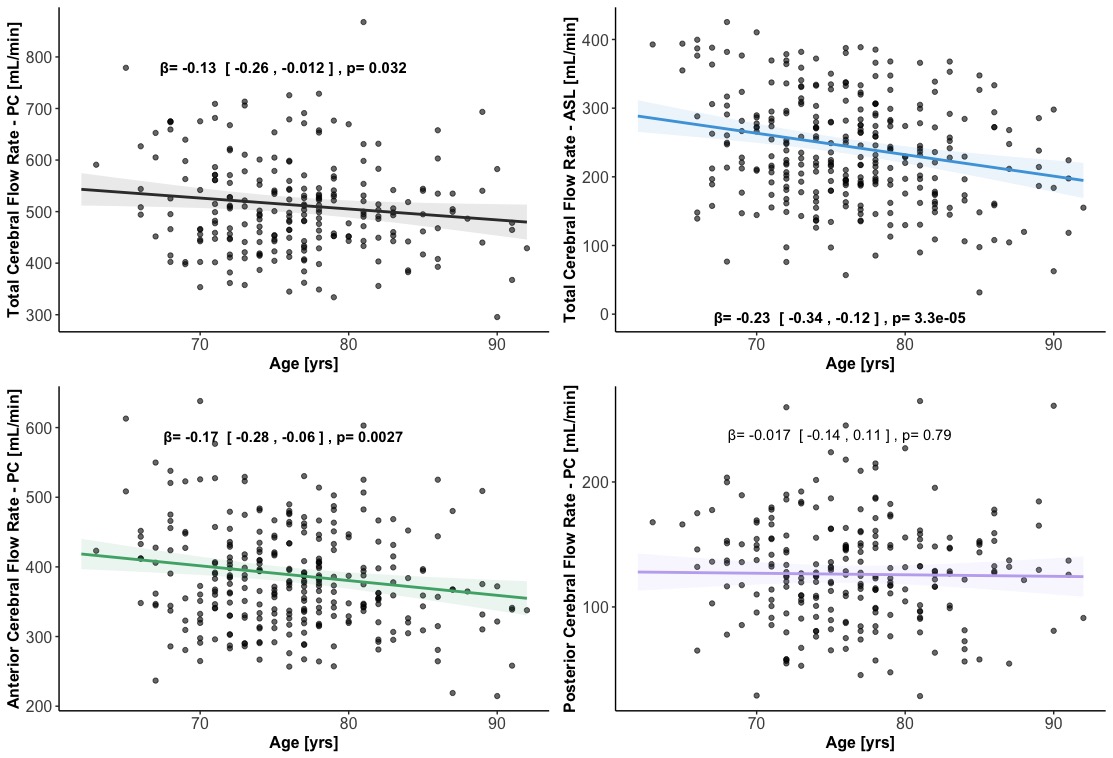


Figure S7: Scatter plots displaying the association between extracranial total (n=232), intracranial total (n=227), extracranial anterior (n=292), and extracranial posterior (n=241) cerebral blood flow and age. Linear regression curves, with 95% confidence intervals, are adjusted for vascular risk factors, intracranial volume, and sex. Scatter plot datapoints are visualized on unadjusted values.
